# Synthesis, predictions of drug-likeness, and pharmacokinetic properties of some chiral thioureas as potent enzyme inhibition agents

**DOI:** 10.55730/1300-0527.3358

**Published:** 2021-09-16

**Authors:** Yusuf SICAK

**Affiliations:** Department of Medicinal and Aromatic Plants, Köyceğiz Vocational School, Muğla Sıtkı Koçman University, Muğla, Turkey

**Keywords:** Thiourea, enzyme inhibition activity, pharmacokinetics, Alzheimer disease, isothiocyanates

## Abstract

A series of chiral thioureas (**1 – 17**) were synthesized from and tested for their anticholinesterase, tyrosinase, and urease enzyme inhibitor activities. Various phenylisothiocyanates were added to solution of l-cysteine in methanol: water (1 : 1 v/v) at room temperature and stirred for 24 h. The precipitated solid was recrystallized from *n*-butanol. Pure compounds were characterized by NMR (^1^H and ^13^C), FTIR, and CHNS. Tertiary amine containing *N*-(4-(diethylamino)phenyl)-*N*′-(2-mercapto-carboxyethanyl)thiourea **17**, *N*-(4-(dimethylamino)phenyl)-*N*′-(2-mercapto-carboxyethanyl)thiourea **16** and trimethoxy containing *N*-(3,4,5-trimethoxyphenyl)-*N*′-(2-mercapto-carboxyethanyl)thiourea **14** were more active than galantamine against AChE and BChE enzymes. In tyrosinase enzyme inhibition activity, compound **14**, **10**, **12**, **6**, **13**, and **11** exhibited higher tyrosinase inhibitory activity showing IC_50_ values of 1.1 ± 0.1, 1.5 ± 0.3, 1.6 ± 0.6, 1.9 ± 0.5, 2.2 ± 0.9 and 2.9 ± 0.2 mM, respectively. In urease enzyme inhibition activity assay, **17** showed higher activity. This work demonstrates the pharmacological significance of chiral thiourea derivatives synthesized from l-cysteine and shows their potential. There is a need to perform more in vitro and in vivo biological activities followed by clinical trials to bring such thiourea to the market.

## 1. Introduction

Expect few ribozymes, enzymes are protein-based biological catalysts. They catalyze chemical reactions in living things under moderate conditions with 100 % efficiency and no by-products. While biochemical transformations are easy and fast in the presence of enzymes, they do not take place, or it takes too long for them to occur in the absence of enzymes. Therefore, enzymes are essential biomolecules for the continuity of life [1]. Enzyme studies are also of great importance in illuminating many problems in our daily life. It is known that some diseases, especially genetic disorders, are caused by a deficiency or complete absence of one or more enzymes. It is also determined that some diseases occur when the enzyme activity is higher than its normal value. For this reason, most drug active ingredients are designed to interact with enzymes.

Enzyme inhibitors are molecules that stop or slow down enzymatic catalysis. The catalysis of enzymes has a very important place in performing cellular activities. Therefore, it is expected for enzyme inhibitors to be of pharmaceutical importance. Enzyme inhibition studies provided valuable information about enzyme mechanisms and helped us illuminate certain metabolic pathways [2].

Many of the drug molecules are inhibitors since inhibition of enzyme activity can correct a metabolic disorder or cause a pathogen to die. Most research in the field of biochemistry and pharmacology has focused on this topic. For the drugs to be considered as enzyme inhibitors, they must be highly specific and act at low concentrations. So, the side effect and toxicity of the drug will be low [3].

The discovery of new enzyme inhibitors is the first step in drug design. One of the ways of discovery and a successful road, which is still used, is the way of trial and error. In this method, drug candidate compounds that are thought to affect the target enzyme are interacted with the enzyme to find the most suitable drug and to develop better derivatives from it [4].

Acetylcholinesterase (AChE, EC 3.1.1.7) and butyrylcholinesterase (BChE, EC 3.1.1.8) are serine-hydrolase enzymes [5]. It has been noticed that both isozymes are found in higher levels in Alzheimer’s disease (AD) [5]. Accordingly, the inhibition of AChE and BChE is considered a significant neuroprotective target in discovery of AD drugs [6]. Urease, an enzyme of family amidohydrolases, is responsible for the urea hydrolysis into ammonia and CO_2_ or carbamate [7,8]. The overexpression of urease leads to various adverse health effects including cryptococcosis, tuberculosis, yersiniosis, peptic ulcers and urolithiasis; thus, inhibition of urease by the potent urease inhibitors has recently attracted scientific attention [8]. The pivotal enzyme of the melanin biosynthesis is tyrosinase. Melanin has primarily a photoprotective role in human skin, while its accumulation may result in skin hyperpigmentation. Therefore, the tyrosinase activity inhibition is considered an interesting way to regulate melanogenesis [9].

Most drugs contain sulfur [10], oxygen, and nitrogen [11]. Biological activity generally increased when both sulfur and nitrogen are present in the same compound. This has increased interest in such compounds in pharmaceutical studies. According to the substituents they contain, thioureas show various biological activities, i.e., anti-HIV, antituberculosis, anticancer, antimalarial, anticonvulsant, anticholinesterase, antityrosinase, and antiurease [7,12–18].

Thioureas are the leading organosulfur compounds used to produce heterocyclics from thiourea skeleton. Thioureas give a good starting for many synthetic drugs [19]. Various aliphatic, aromatic, heterocyclic, and chiral thioureas can be synthesized from isothiocyanates and primary or secondary amines by condensation in various solvents. They are reported for their pharmaceutical, antitubercular, antiinflammatory, anticonvulsant, anticancer, anti-thyroid, anthelmintic, anti-HIV, high-density lipoprotein (HDL)-raising, antidiabetic, anti-hypertensive, anti-epileptic, DNA-binding, hypnotic, and anesthetic activities [20–27]. It can also be used as an enzyme inhibitor such as acetylcholinesterase, butyrylcholinesterase, anti-phenoloxidase, carbonic anhydrase, etc. [19,28–31].

In recent years, due to the rapid increase in the studies on the development of chiral drug active substances, chiral drugs have become a subject of interest in the pharmaceutical industry. However, asymmetric synthesis is used for obtaining single enantiomers, but it is difficult to find suitable reagents and starting materials. The most suitable method is to use chiral catalysts or kinetic separation of the racemic mixture using enzymes, which has limitations. As a result, the separation of these substances into their enantiomers is a very difficult process because they are obtained as racemic mixtures. In addition, it has been observed that the toxic values of the product formed because completely synthetic reactions are high. Chiral thiourea and their derivatives show many biological activities such as anticancer, anticonvulsant, antibacterial, anti-HIV, antifungal, antiviral [32].

This study was aimed to synthesize new biological active chiral thioureas starting from l-cysteine and isothiocyanates and to check their cholinesterase (ChE), tyrosinase, and urea inhibition activities. Substituent effect on the biological activity was also studied that may lead to the design of new productive drugs. Thiourea derivatives derived from various aromatic isothiocyanates have been reported for their novel pharmacological activities such as in the treatment of Alzheimer’s disease, pigmentation of melanin, and diseases that may arise from *Helicobacter pylori*. Thus, it is expected that the synthesized organic compounds may be new generation drugs having these properties.

## 2. Experimental

### 2.1. Chemistry

The solvents and chemicals used in this study were obtained from Aldrich, Fluka, Merck. The thin layer chromatography plates (TLC, Merck Silica Gel 60 F254) were used to monitor the reactions. Melting points of the synthesized compounds were controlled by a Stuart SMP20 automated melting point apparatus (UK). Infrared spectra were recorded on a PerkinElmer 1620 model FT-IR spectrophotometer while elemental analyses (C, H, N, S) were performed on a VarioMICRO elemental analyzer (Elemental Analyses System, GmbH, Hanau, Germany). ^1^H NMR spectra were run on a Bruker Avance-DPX-400 NMR spectrometer (Bruker BioSpin, Billerica, MA, USA) in DMSO-*d_6_* solvent where tetramethylsilane (TMS) was used as an internal standard. All biological measurements were carried out on SpectraMax 340PC384 (Molecular Devices, San Jose, CA, USA) equipment using a 96-well microplate reader.

#### 2.1.1. General procedure of compounds (1–17)

Various phenylisotiyocyanates (1 mmol) were added to the solution of l-cysteine (1 mmol) in methanol : water (1 : 1 v :v) at room temperature, and the mixture was stirred for 24 h. The precipitated solid was recrystallized from *n*-butanol and submitted for structural elucidation.

##### N-phenyl-N′-(2-mercapto-carboxyethanyl)thiourea (1) [33]

White solid. Yield: 57 %. mp 188–190 °C. IR ν_max_ (cm^−1^): 3676 (OH), 3261, 3227 (N - H), 2971 (Ar - CH), 2901 (R - CH), 2509 (C - S), 1587 (C = O), 1492 (N - H), 1359 (C = S), 1229 (C - N). Anal. Calcd for C_10_H_12_N_2_O_2_S_2_: C 46.85; H 4.72; N, 10.93; S, 25.02 %. Found: C 46.22; H 4.70; N 10.13; S 24.88 %.

##### N-(4-bromophenyl)-N′-(2-mercapto-carboxyethanyl)thiourea (2)

White solid. Yield: 51 %. mp 200–201 °C. IR ν_max_ (cm^−1^): 3671 (OH); 3082, 3003 (N - H), 2988 (Ar - CH), 2901 (R - CH), 2601 (C - S), 1587 (C = O), 1477 (N - H), 1399 (C = S), 1243 (C - N), 1066 (C - Br). Anal. Calcd for C_10_H_11_BrN_2_O_2_S_2_: C 35.83; H 3.31; N 8.36; S 19.13 %. Found: C 35.77; H 3.24; N 8.21; S 19.09 %.

##### N-(4-chlorophenyl)-N′-(2-mercapto-carboxyethanyl)thiourea (3) [33]

White solid. Yield: 45 %. mp 193–195 °C. IR ν_max_ (cm^−1^): 3676 (OH), 3158, 3112 (N-H), 2987 (Ar - CH), 2901 (R - CH), 2551 (C - S), 1594 (C = O), 1488 (N - H), 1348 (C=S), 1261 (C-N), 1096 (C - Cl). Anal. Calcd for C_10_H_11_ClN_2_O_2_S_2_: C 41.30; H 3.81; N 9.63; S 22.05 %. Found: C 41.03; H 3.76; N 9.55; S 22.01 %.

##### N-(4-fluorophenyl)-N′-(2-mercapto-carboxyethanyl)thiourea (4)

White solid. Yield: 61 %. mp 181–182 °C. IR ν_max_ (cm^−1^): 3663 (OH), 3195, 3103 (N - H), 2987 (Ar - CH), 2901 (R - CH), 2582 (C - S), 1595 (C = O), 1505 (N - H), 1340 (C = S), 1240 (C - N), 1223 (C - F). Anal. Calcd for C_10_H_11_FN_2_O_2_S_2_: C 43.78; H 4.04; N 10.21; S 23.38 %. Found: C, 43.40; H, 4.01; N, 10.15; S, 23.29 %.

##### N-(4-(trifluoromethyl)phenyl)-N′-(2-mercapto-carboxyethanyl)thiourea (5)

White solid. Yield: 24 %. mp 205–206 °C. IR ν_max_ (cm^−1^): 3676 (OH), 3212, 3190 (N - H), 2976 (Ar - CH), 2901 (R - CH), 2591 (C - S), 1600 (C = O), 1516 (N - H), 1319 (C=S), 1264 (C - N), 1208 (C - F). Anal. Calcd for C_11_H_11_F_3_N_2_O_2_S_2_: C 40.73; H 3.42; N 8.64; S 19.77 %. Found: C 40.66; H 3.31; N 8.61; S 19.53 %.

##### N-(4-methoxyphenyl)-N′-(2-mercapto-carboxyethanyl)thiourea (6)

White solid. Yield: 51 %. mp 211–213 °C. IR ν_max_ (cm^−1^): 3663 (OH), 3256, 3197 (N - H), 2989 (Ar - CH), 2907 (R - CH), 2564 (C-S), 1587 (C = O), 1502 (N - H), 1360 (C = S), 1296 (C - N). Anal. Calcd for C_11_H_14_N_2_O_3_S_2_: C 46.14; H 4.93; N 9.78; S 22.39 %. Found: C 46.11; H 4.80; N 9.66; S 22.24 %.

##### N-(4-cyanophenyl)-N′-(2-mercapto-carboxyethanyl)thiourea (7)

White solid. Yield: 84 %. mp 217–219 °C. IR ν_max_ (cm^−1^): 3676 (OH), 3227, 3177 (N - H), 3065 (Ar - CH), 2968 (R - CH), 2568 (C - S), 1615 (C = O), 1505 (N - H), 1334 (C = S), 1297 (C - N). Anal. Calcd for C_11_H_11_N_3_O_2_S_2_: C 46.96; H 3.94; N 14.93; S 22.79 %. Found: C 46.69; H 3.82; N 14.90; S 22.66 %.

##### N-(5-chloro-2-methoxyphenyl)-N′-(2-mercapto-carboxyethanyl)thiourea (8)

White solid. Yield: 42 %. mp 209–210 °C. IR ν_max_ (cm^−1^): 3663 (OH), 3274, 3075 (N - H), 2973 (Ar - CH), 2901 (R - CH), 2543 (C - S), 1591 (C = O), 1488 (N - H), 1405 (C = S), 1293 (C - N), 1015 (C - Cl). Anal. Calcd for C_11_H_13_ClN_2_O_3_S_2_: C 41.18; H 4.08; N 8.73; S 19.99 %. Found: C, 41.10; H, 4.00; N, 8.71; S, 19.86 %.

##### N-(2-chloro-5-(trifluoromethyl)phenyl)-N′-(2-mercapto-carboxyethanyl)thiourea (9)

White solid. Yield: 28 %. mp 247–248 °C. IR ν_max_ (cm^−1^): 3659 (OH), 3251, 3201 (N - H), 2961 (Ar - CH), 2928 (R - CH), 2531 (C - S), 1670 (C = O), 1522 (N - H), 1324 (C = S), 1294 (C - N), 1081 (C - Cl). Anal. Calcd for C_11_H_10_ClFN_2_O_2_S_2_: C 36.82; H 2.81; N 7.81; S 17.87 %. Found: C 36.76; H 2.73; N 7.64; S, 17.77 %.

##### N-(2,4-dimethoxyphenyl)-N′-(2-mercapto-carboxyethanyl)thiourea (10)

White solid. Yield: 11 %. mp 240–241 °C. IR ν_max_ (cm^−1^): 3672 (OH), 3339, 3148 (N - H), 3148 (Ar - CH), 2968 (R - CH), 2524 (C - S), 1596 (C = O), 1502 (N - H), 1384 (C = S), 1284 (C - N). Anal. Calcd for C_12_H_16_N_2_O_4_S_2_: C 45.55; H 5.10; N 8.85; S 20.27 %. Found: C 45.28; H 5.05; N 8.74; S 20.21 %.

##### N-(2,5-dimethoxyphenyl)-N′-(2-mercapto-carboxyethanyl)thiourea (11)

White solid. Yield: 64 %. mp 222–223 °C. Anal. Calcd for C_12_H_16_N_2_O_4_S_2_: C 45.55; H 5.10; N 8.85; S 20.27 %. Found: C, 45.52; H, 5.07; N, 8.63; S, 20.18 %.

##### N-(3,4-dimethoxyphenyl)-N′-(2-mercapto-carboxyethanyl)thiourea (12)

White solid. Yield: 84 %. mp 229–231 °C. IR ν_max_ (cm^−1^): 3676 (OH), 3256, 3214 (N - H), 2988 (Ar - CH), 2908 (R - CH), 2516 (C - S), 1612 (C = O), 1511 (N - H), 1393 (C = S), 1267 (C - N). Anal. Calcd for C_12_H_16_N_2_O_4_S_2_: C 45.55; H 5.10; N 8.85; S 20.27 %. Found: C 44.98; H 5.03; N 8.84; S 20.10 %.

##### N-(3,5-dimethoxyphenyl)-N′-(2-mercapto-carboxyethanyl)thiourea (13)

Orange solid. Yield: 32 %. mp 230–232 °C. IR ν_max_ (cm^−1^): 3670 (OH), 3316, 3083 (N - H), 3082 (Ar - CH), 2987 (R - CH), 2544 (C - S), 1588 (C = O); 1537 (N - H); 1420 (C=S); 1257 (C-N). Anal. Calcd for C_12_H_16_N_2_O_4_S_2_: C 45.55; H 5.10; N 8.85; S 20.27 %. Found: C 45.41; H 5.02; N 8.80; S 20.13 %.

##### N-(3,4,5-trimethoxyphenyl)-N′-(2-mercapto-carboxyethanyl)thiourea (14)

White solid. Yield: 27 %. mp 239–240 °C. IR ν_max_ (cm^−1^): 3672 (OH), 3193, 3138 (N - H), 2989 (Ar - CH), 2969 (R - CH), 2532 (C - S), 1601 (C = O), 1504 (N - H), 1368 (C = S), 1228 (C - N). Anal. Calcd for C_13_H_18_N_2_O_5_S_2_: C 45.07; H 5.24; N 8.09; S 18.51 %. Found: C 45.03; H 5.11; N 8.04; S 18.30 %.

##### N-(2,5-dichlorophenyl)-N′-(2-mercapto-carboxyethanyl)thiourea (15)

White solid. Yield: 35 %. mp 233–234 °C. IR ν_max_ (cm^−1^): 3676 (OH), 3187, 3105 (N - H), 2988 (Ar - CH), 2901 (R - CH), 2511 (C - S), 1583 (C =O), 1507 (N - H), 1384 (C=S), 1295 (C - N), 1184 (C - S). Anal. Calcd for C_10_H_10_Cl_2_N_2_O_2_S_2_: C 36.93; H 3.10; N 8.61; S 19.72 %. Found: C 36.90; H 3.04; N 8.53; S 19.61 %.

##### N-(4-(dimethylamino)phenyl)-N′-(2-mercapto-carboxyethanyl)thiourea (16) [34]

Yellow solid. Yield: 74 %. mp 238–239 °C. IR ν_max_ (cm^−1^): 3676 (OH), 3252, 3178 (N-H), 2988 (Ar-CH), 2901 (R-CH), 2528 (C-S), 1591 (C=O), 1514 (N-H), 1347 (C=S), 1295 (C-N). Anal. Calcd for C_12_H_17_N_3_O_2_S_2_: C 48.14; H 5.72; N 14.03; S 21.42 %. Found: C 48.10; H 5.67; N 14.03; S 21.34 %.

##### N-(4-(diethylamino)phenyl)-N′-(2-mercapto-carboxyethanyl)thiourea (17)

White solid. Yield: 70 %. mp 225–226 °C. IR ν_max_ (cm^−1^): 3676 (OH), 3357, 3204 (N - H), 2969 (Ar - CH), 2900 (R - CH), 2537 (C - S), 1606 (C = O), 1515 (N - H), 1401 (C = S), 1264 (C - N), 1184 (C - S). Anal. Calcd for C_14_H_21_N_3_O_2_S_2_: C 51.35; H 6.46; N 12.83; S 19.58 %. Found: C 51.22; H 6.39; N 12.77; S 19.51 %.

### 2.2. Biological activities

#### 2.2.1. Anticholinesterase inhibitory activity

The in vitro anticholinesterase activity of all synthesized compounds (**1 – 17**) was performed according to Ellman’s method using 96 well microplate reader. Herein, acetylcholinesterase (AChE) from electric eel and butyrylcholinesterase (BChE) horse serum was used. The acetylthiocholine iodide and butyryl thiocholine chloride were utilized as substrates. DTNB (5,50-dithiobis(2-nitrobenzoic) acid was used as coloring agent to measure the anticholinesterase activity [35]. The compounds (**1 – 17**) were tested at four μM concentrations, i.e., 400-200–100-50 μM in triplicate measurements.

#### 2.2.2. Tyrosinase inhibitory activity

The solutions of chiral thiourea compound (**17**) were prepared at four different concentrations, i.e., 400, 200, 100, and 50 mM in EtOH. Additionally, EtOH was used as a control, while kojic acid with l-mimosine was used as tyrosinase standards. The results are given as 50 % concentration (IC_50_). The spectrophotometric analysis of tyrosinase inhibitory activities was performed according to the slightly modified literature procedures of Hearing [36].

#### 2.2.3. Urease inhibitory activity

Solutions of chiral thioureas (**1 – 17**) were prepared at four different concentrations, i.e., 400, 200, 100, and 50 μM for urease inhibitory assay in EtOH. Additionally, EtOH was used as a control, while thiourea was used as urease standards. The results are given as 50 % concentration (IC_50_) for urease inhibitory activity assay. The spectrophotometric analysis of urease inhibitory activity was performed according to the literature procedures by measuring ammonia production using the indophenol method as described earlier [37].

### 2.3. In silico ADME prediction

Computational studies of the synthesized thioureas **1 – 17** were predicted using Molinspiration for molecular properties, Molsoft for absorption of molecular properties, and SwissADME online [38] server. Calculated molecular volume (Mv), molecular weight (Mw), the logarithm of the partition coefficient (milog P), the number of hydrogen-bond donors (nOHNH), the number of hydrogen-bond acceptors (nOH), topological polar surface area (TPSA), the number of rotatable bonds (Nrotb), and Lipinki’s rule of five were determined using Molinspiration and Molsoft online property calculation toolkits. The percentage absorption was calculated by the equation [38]:

### 2.4. Statistical analysis

The bioactivity tests were carried out in triplicate at four different concentrations. Results are given as IC_50_ (μg/mL). The data were the mean ± S.E.M. (standard error of the meaning) of triplicate analyses of each concentration under the 95 % reliability (*p* < 0.05).

## 3. Results and discussion

**Figure 1:**
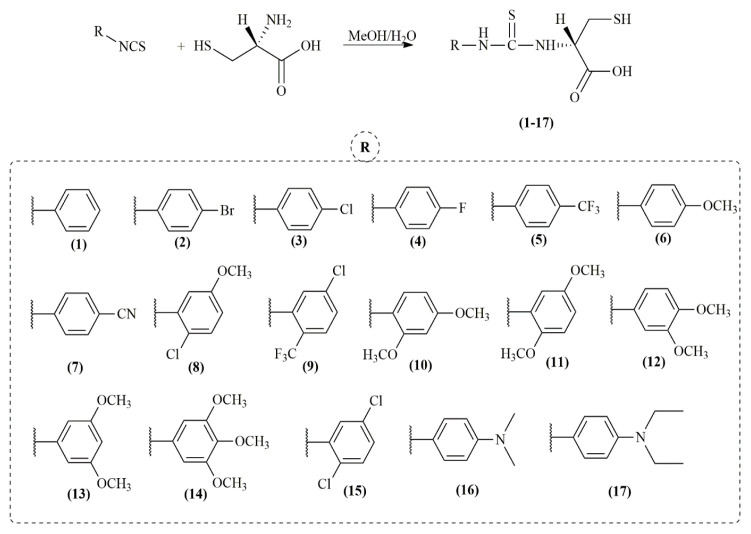
Synthesis of l-cysteine-based thioureas tuned with various functionalities.

A series of chiral thioureas **(1 – 17)** derived from l-cysteine were synthesized in this study. While compounds **1** [33], **3** [33], and **16** [34] from the synthesized target are known substances, the others are novel. The preparation of the target molecules was carried out by synthetic route outlined in Figure 1.

In the IR spectra of chiral thioureas **(1 – 17)** (compound **11** except), a weak band was observed in compound **11** at 3082–3357 cm^−1^ for NH bands attached to the aromatic ring and at 3003–3227 cm^−1^ for NH bands attached to aliphatic - CH - as expected. Additionally, the -OH band of the carboxylic acid group at 3659 – 3676 cm^−1^, aromatic C - H bands at 2961 – 3148 cm^−1^, aliphatic C - H bands at 2900 – 2969 cm^−1^, C = O band of the carbonyl group at 1568 – 1670 cm^−1^, NH band at 1477 – 1537 cm^−1^, C = S band of thiocarbonyl group at 1319 – 1420 cm^−1^, C - N band at 1228 – 1297 cm^−1^ and SH band of thiol group at 2509–2601 cm^−1^ were observed. The characteristic N - H, C = S and C - N tensile vibrations has been reported at 3190–3384, 1302–1393 and 1201–1282 cm^−1^, respectively [14]. C - F band of compound **4**, **5** and **9** containing fluorine were observed at 1208–1223 cm^−1^, C - Cl band of compound **5**, **8** and **9** containing chlorine at 1081–1215 cm^−1^ while C - Br band of compound **2** containing bromine were observed at 1066 cm^−1^.

In the ^1^H NMR spectra of the synthesized compounds **(1 – 17)**, -NH peaks linked to the aromatic ring of the thiourea ( - NH - CS - NH - ) group were observed at 7.13–9.61 ppm, while the - NH peaks linked to the aliphatic structure were observed at 7.71–10.55 ppm. Proton of carboxylic acid ( - COOH) was observed at 9.70–11.72 ppm; - CH - proton as multiple at 4.78–5.38 ppm; and the thiol ( - SH) proton were found at 1.08–3.17 ppm. - CH_2_ - protons resonated as Ha and Hb at 2.91–3.85 and 2.81–3.64 ppm, respectively. Protons in the - C*H*_2_ - SH are neighboring the chiral carbon, which are heterotopic as reported in our previously study [39]. Detailed ^1^HNMR peaks of all the synthesized compounds are given in Table 1.

All the synthesized compounds were tested for their in vitro anticholinesterase inhibitory activity against AChE and BChE enzymes. The results are compared with galantamine as given in Table 2. Among the synthesized series, **17**, **16** and **14** exhibited excellent activities than galantamine in both assays. Moreover, against AChE, **17** (IC_50_: 5.7 ± 1.0 μM), **10** (IC_50_: 6.8 ± 1.1 μM), **9** (IC_50_: 7.2 ± 0.5 μM), **12** (IC_50_: 7.8 ± 0.6 μM), **6** (IC_50_: 8.1 ± 0.9 μM), and **8** (IC_50_: 9.5 ± 1.2 μM) compounds exhibited higher activities. In the BChE inhibitory assay, **17** (IC_50_: 18.1 ± 0.5 μM), **16** (IC_50_: 24.6 ± 0.7 μM), **14** (IC_50_: 29.5 ± 1.1 μM), **13** (IC_50_: 37.2 ± 0.1 μM) and **10** (IC_50_: 45.8 ± 0.4 μM) displayed higher activities than galantamine (IC_50_: 46.4 ± 0.8 μM).

Synthesized chiral thiourea derivatives were evaluated for their inhibitory effects on tyrosine enzyme at different concentrations. The results were compared with kojic acid and l-mimosine as given in Table 2. According to assay results, **14**, **10**, **12**, **6**, **13**, and **11** showed the best tyrosinase inhibitory activity with IC_50_ values of 1.1 ± 0.1, 1.5 ± 0.3, 1.6 ± 0.6, 1.9 ± 0.5, 2.2 ± 0.9 and 2.9 ± 0.2 mM, respectively.

The chiral thiourea derivatives were evaluated for their inhibitory effects on urease enzyme at different concentrations. The results were compared with thiourea (Table 2). Compound **14** (IC_50_: 13.4 ± 0.8 μM), **17** (IC_50_: 16.5 ± 0.6 μM), **10** (IC_50_: 20.9 ± 1.0 μM), and **11** (IC_50_: 22.1 ± 0.1 μM) exhibited excellent activities in the urease inhibitory activity than thiourea with IC_50_ of 24.20 ± 0.3 μM.

In the development the oral bioavailability of new drug candidates, obtaining the maximum level of bioavailability is thought to play a significant role [40, 41]. Many drug candidates have a poor pharmacokinetic profile that limits their development. The ADME properties of chiral thioureas derivatives **(1 – 17)** were computed using SwissADME online toolkit. Evaluated parameters are presented in Table S1. Lipinski’s rule of five is an important approach to describe the relationships between physicochemical and pharmacokinetic properties in particularly and to be satisfied. According to this theory, this *in silico* study determines the drug-likeness of synthesized compounds compared to known drug. In Table S1, among synthesized chiral-thiourea drug-likeness scores was higher (−0.07) for **14**. A good oral bioavailability is evaluated as the main of Log P ( < 5), MW ( < 500), HBA ( ≤ 10) and HBD ( < 5) values. The molecular flexibility is identified by the number of rotatable bonds (nROTB) which must be <10. nROTB values for the synthesized chiral derivatives are in the range of 6–9. Solubility parameter is whether the drug is soluble or moderately soluble, an important factor for the absorption of the drug. The insolubility is calculated using log S (ESOL) ranges 0–6 [42]. Pharmacokinetic properties such as blood brain barrier (BBB) permeability, gastrointestinal absorption and skin permeability are presented in Table S2. The bioavailability scores are given in Table S2 as Lipinski rule, Ghose (Amgen), Veber (GSK), Egan (Pharmacia) and Muegge (Bayer). P-glycoprotein (P - gp substrate) plays a prominent role in absorption and disposition of drugs. CYP1A2, CYP2C19, CYP2C9, CYP2D6, CYP3A4, which are the inhibitors of isoenzyme, means there might be the opportunities of accumulation or drug-drug interaction causing toxicity. According to the Ghose rule (put forward by Amgen),−0.4–+ 5.6 of log P value should be between the 40^−1^30 of molar refractivity, 180–480 of molecular weight and 20–70 of number of atoms. According to the Egan rule, which put forward by Pharmacia Company for the prediction of human intestinal absorption stating, the log P value should not be more than 5.88 while TPSA should not be more than 131.6. The orally active stated by Verber for GSK pharmaceuticals, particularly, in some drugs like steroids, the molecular weight should be more than 500 D and nROTB number 10 or fewer, while PSA should not be more than 140 A0. Parameters of the Muegge rule proposed by Bayer Pharmaceuticals should be Mw (200–600 D), log P (−2–+5), TPSA ( < 150), nROTB ( < 15), HBD ( < 5), and HBA ( > 10) and, < 7 number of rings, > 4 number of carbon atoms, and number of heteroatoms more than 1. PAINS in Table S2, indicated whether the compound was a specific condition inherently. Synthetic Accessibility (SA) Score attributed principally on the assumption that the molecule in ‘really’ attainable having correlation with the ease of synthesis [42]. The bioactivity scores by a numerical value computed by Molinspiration of synthesized chiral - thioureas were offered as comparing with standard drug based on enzyme inhibitor (EI), GPCR ligand (GPCRL), ion channel modulator (ICM), kinase inhibitor (KI), nuclear receptor legend (NRL) and protease inhibitor (PI), as given in Table S3.

These studies give us important information without conducting experimental studies on the possible effects of chemical compounds on metabolism from their molecular structure and whether they can be used as drugs. Thousands of molecules are synthesized in the world every year. If will be very costly, is we do their bioactivity tests in the laboratory environment. Therefore, ADME studies are very important in understanding the potential of compounds as drugs [43]. It plays an important role in the drug development process, which lessens pharmacokinetic failures in the optimization stage of lead molecules and studies in several clinical phases of drug candidates with known pharmacodynamic and pharmacokinetic properties. This strategy is an efficient alternate approach to *in silico* prediction to use ADME (Absorption, Distribution, Metabolism and Elimination) prediction that brings us advantages over experimental predictions. For this purpose, the ADME studies of synthesized chiral thiourea compounds were carried out and are shown in Figure S1. Reliable drug considering bioavailability radar head, flexibility ( < 9), lipophilicity (−0.7–+ 0.5), polarity (20A^−1^30A), saturation (0.25 < 1), size (150 g/mol > 500g), and solubility ( < 6).

**Figure 2:**
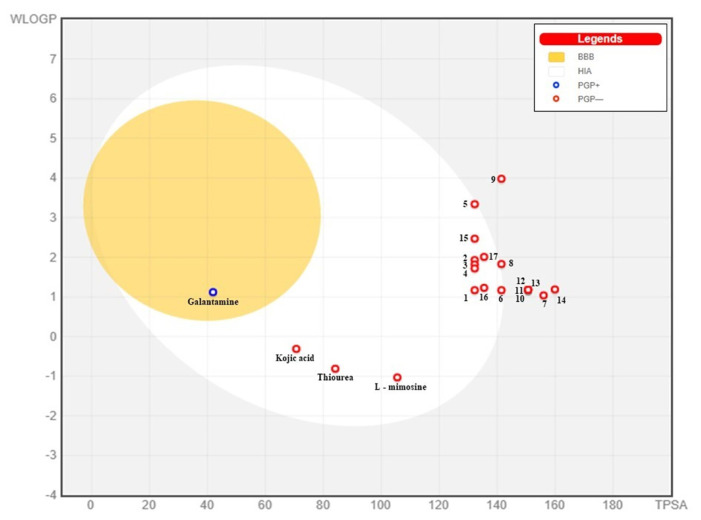
Graphical distribution of synthesized chiral thioureas and enzyme inhibitor standards according to the BOILED - EGG predictive model.

Gastrointestinal absorption (GI) and blood-brain barrier (BBB) are two pharmacokinetic behaviors critical to predict at different stages of the processes of drug discovery. As an accurate predictive model, the Brain Or IntestinaL EstimateD permeation (BOILED - Egg) method is handled by calculating the polarity and lipophilicity of small molecules. This approach is widely used to provide a visual cue in profiling synthesis of novel compounds in terms of their potential to be orally absorbed [44, 45]. For drug discovery, an attempt was made to evaluate the predictive power of the model for gastrointestinal passive absorption, given the undeniable efficacy of the Egan egg, and to describe it by predicting access to the brain *via* passive diffusion to finally place the BOILED - Egg (Brain or Gut Prediction model of Change). The graphical prediction of GI and BBB permeation of the synthesized thiourea compounds is shown in Figure 2. It explains well penetration within the brain with good intestinal absorption for yellow region, intestinal absorption for white region, and poor intestinal absorption for gray region. According to the BOILED - Egg plot, none of the chiral thiourea for BBB representing the yellow circle found in this region. In the white ellipse, which is the human intestinal absorption, compounds (compound **7, 10, 11, 12, 13**, and **14** except) lie in this white ellipse. Compounds **7, 10, 11, 12, 13**, and **14** were in the gray area showing poor intestinal absorption. The blue dots, evidence of exhibiting good bioavailability, indicated that compounds could be a substrate for P - glycoprotein, reducing its absorption and penetration within brain. Particularly, compounds **1, 2, 3, 4, 5, 15, 16** and **17** may be promising agents that can be absorbed very easily by the gastrointestinal tract without a potential BBB permeability.

Alzheimer’s is an irreversible brain disorder defined by the loss of memory and learning ability in older patients. This disease is influencing large population around the world. Most of the clinically used drugs used to treat Alzheimer’s disease are acetylcholinesterase inhibitors (AChEIs). The enzyme Acetylcholinesterase (AChE), (E.C.3.1.1.7) have a key role in the hydrolysis of the released acetylcholine neurotransmitter [46]. If acetylcholinesterase is hindered, the hydrolysis of acetylcholine can be inspected which can be beneficial in the Alzheimer’s or dementia symptomatic relief [47]. But these drugs can maintain only symptomatic benefits and suffer with therapeutic potential loss in time. Besides, there is an immediate need of novel cholinesterase inhibitor agents with force and active therapeutic for the Alzheimer’s treatment. The in vitro anticholinesterase, tyrosinase and urease inhibition activities of the synthesized thiourea compounds **(1 – 17)** is being reported in this study for the first time. Generally, these compounds exhibited excellent anticholinesterase, tyrosinase and urease inhibition activities. Tertiary amine (**17**, **16**) and trimethoxy (**14**) based compounds showed more activity than galantamine against both AChE and BChE enzymes. According to the obtained data, the chiral thiourea derivatives containing a tertiary amine and trimethoxy groups utilized for their AChE and BChE inhibitory activity may be promising for further studies to treat Alzheimer’s disease.

Melanin is a group of natural pigments that plays primary role in determining the color of eye, hair, and skin. Congenital tyrosinase deficiency causes melanin production disorder in the body while skin defects happen due to excess melanin synthesis. Cancer and Parkinson’s disease depends on abnormalities in activity of tyrosinase [48–51]. In this context, many researchers that modulate the tyrosinase activity have discovered numerous natural and synthetic compounds [52–56]. Especially, synthetic phenylthiourea derivatives comprise another well-known major class of tyrosinase inhibitors. Among the synthesized compounds that thiourea derived compounds containing methoxy group had better tyrosinase activity. In general, it was found that compounds showed better tyrosinase activity as the number of methoxy groups increased in the structure. In this context, it can be concluded that the synthesis of different thiourea derivatives containing methoxy groups may be potential candidates for the treatment of skin disease associated with melanin biosynthesis.

Urease (urea amidohydrolase, E.C. 3.5.1.5) is a nickel-containing enzyme that catalyzes the urea hydrolysis into ammonia and carbamate, which is the last step of nitrogen metabolism in living organisms [57, 58]. The quick and spontaneous decomposition of carbamate gives carbonic acid and a second molecule of ammonia. These reactions may lead to significant pH increase. Moreover, they are liable for negative impacts of urease activity in animal and human health. Urease is one of the most common factors that is responsible for gastrointestinal infections and urinary tract [59]. Urease has direct influence on the infectious stone formation. Furthermore, it is involved in the pathogenesis of ammonia and hepatic encephalopathy, urolithiasis, pyelonephritis, hepatic coma, and urinary catheter encrustation [60, 61]. It is also known as the main reason of pathologies induced by *Helicobacter pylori* (HP), which permits bacteria to survive at acidic pH of the stomach during colonization and consequently plays a significant role in the gastric and peptic ulcer pathogenesis [45].

## 4. Conclusion

A series of chiral *N*-(substitutedphenyl)-*N*′-(2-mercapto-carboxyethanyl)thioureas derivatives **(1–17)** were synthesized in this study. Structures of synthesized compounds were confirmed by spectroscopic methods using IR, ^1^H NMR, and elemental analysis (C, H, N, S). Compounds containing tertiary amine in the synthesized thiourea derivatives, especially those containing 2,4-diomethoxy, 2,5-dimethoxy, and 3,4,5-trimethoxy, showed notable urease activities. In conclusion, novel thiourea-derived pharmaceuticals can be synthesized for urease inhibition based on tertiary amine and methoxy groups.

Figure S1 The bioavailability radar of synthesized chiral thioureas and enzyme inhibitor standards using Swiss ADME predictor.

**Table S1:** SMILES, Lipinski rule of five and drug likeness of the synthesized thioureas.

Thioureas	SMILES	Molecular properties	Drug likeliness
**1**	C1=CC=CC(=C1)N(C(N(C(C(=O)O[H])CS[H])[H])=S)[H]	Molecular formula: C_10_H_12_N_2_O_2_S_2_ Molecular weight: 256.03 Number of HBA: 4 Number of HBD: 4 MolLogP: 1.12 MolLogS: ^−1^.95 (in Log (moles/L)) 2865.75 (in mg/L) MolPSA: 48.69 A^2^ MolVol: 225.28 A^3^ Number of stereo centers: 1	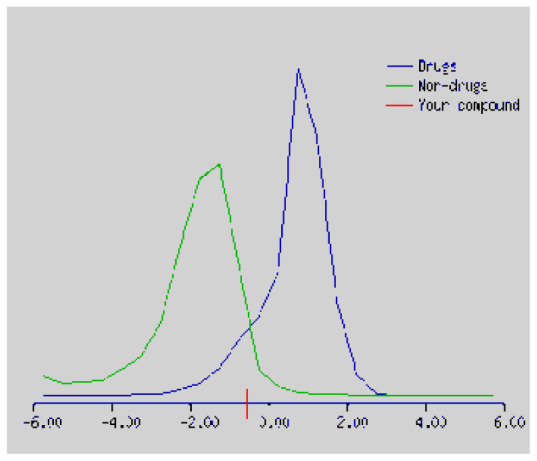 Drug-likeness model score: −0.56
**2**	C1=C(C=CC(=C1)N(C(N(C(C(=O)O[H])CS[H])[H])=S)[H])Br	Molecular formula: C_10_H_11_BrN_2_O_2_S_2_ Molecular weight: 333.94 Number of HBA: 4 Number of HBD: 4 MolLogP: 2.15 MolLogS: −2.76 (in Log (moles/L)) 584.03 (in mg/L) MolPSA: 48.69 A^2^ MolVol: 247.13 A^3^ Number of stereo centers: 1	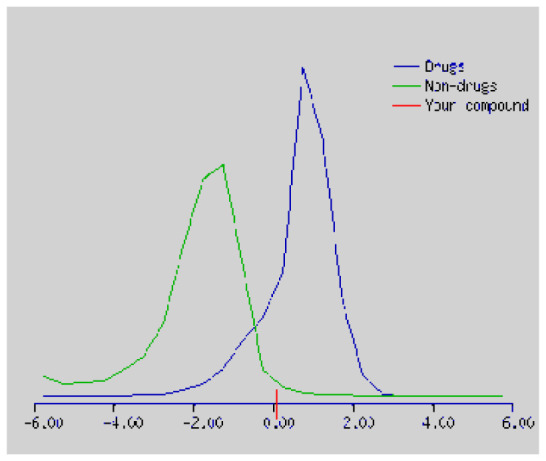 Drug-likeness model score: 0.09
**3**	C1=C(C=CC(=C1)N(C(N(C(C(=O)O[H])CS[H])[H])=S)[H])Cl	Molecular formula: C_10_H_11_ClN_2_O_2_S_2_ Molecular weight: 290.00 Number of HBA: 4 Number of HBD: 4 MolLogP: 1.91 MolLogS: −2.66 (in Log (moles/L)) 632.30 (in mg/L) MolPSA: 48.69 A^2^ MolVol: 242.47 A^3^ Number of stereo centers: 1	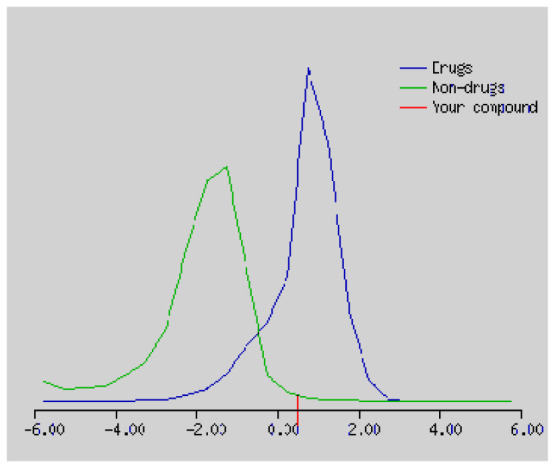 Drug-likeness model score: 0.48
**4**	C1=C(C=CC(=C1)N(C(N(C(C(=O)O[H])CS[H])[H])=S)[H])F	Molecular formula: C_10_H_11_FN_2_O_2_S_2_ Molecular weight: 274.02 Number of HBA: 4 Number of HBD: 4 MolLogP: 1.38 MolLogS: ^−1^.99 (in Log (moles/L)) 2782.27 (in mg/L) MolPSA: 48.69 A^2^ MolVol: 231.19 A^3^ Number of stereo centers: 1	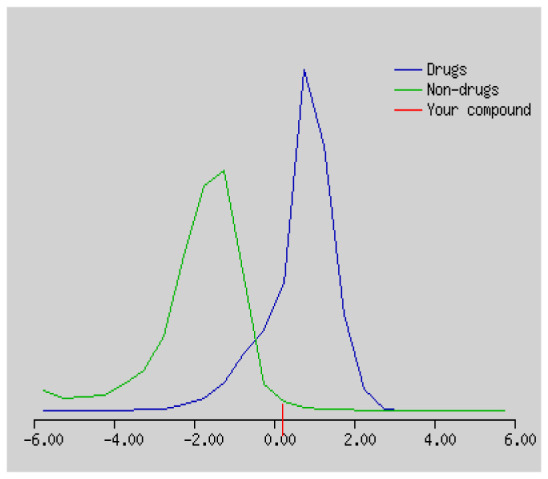 Drug-likeness model score: 0.23
**5**	C1=C(C=CC(=C1)N(C(N(C(C(=O)O[H])CS[H])[H])=S)[H])C(F)(F)F	Molecular formula: C_11_H_11_F_3_N_2_O_2_S_2_ Molecular weight: 342.02 Number of HBA: 4 Number of HBD: 4 MolLogP: 2.26 MolLogS: −2.80 (in Log (moles/L)) 516.91 (in mg/L) MolPSA: 48.69 A^2^ MolVol: 262.20 A^3^ Number of stereo centers: 1	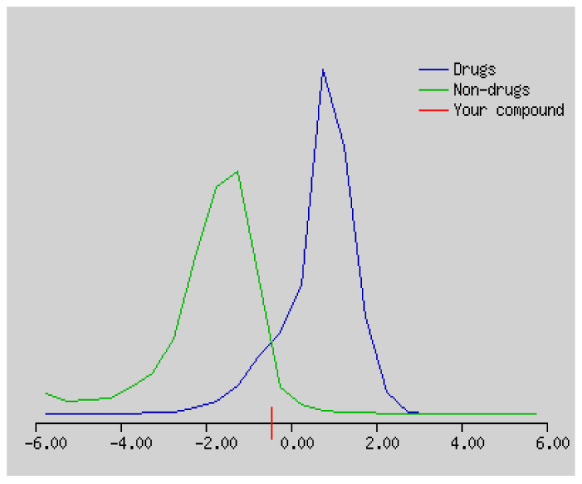 Drug-likeness model score: −0.44
**6**	C1=C(C=CC(=C1)N(C(N(C(C(=O)O[H])CS[H])[H])=S)[H])OC	Molecular formula: C_11_H_14_N_2_O_3_S_2_ Molecular weight: 286.04 Number of HBA: 5 Number of HBD: 4 MolLogP: 0.37 MolLogS: ^−1^.85 (in Log (moles/L)) 4010.36 (in mg/L) MolPSA: 56.24 A^2^ MolVol: 262.12 A^3^ Number of stereo centers: 1	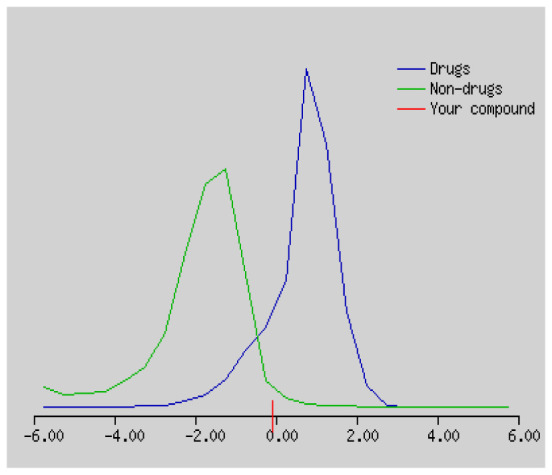 Drug-likeness model score: −0.08
**7**	C1=C(C=CC(=C1)N(C(N(C(C(=O)O[H])CS[H])[H])=S)[H])C#N	Molecular formula: C_11_H_11_N_3_O_2_S_2_ Molecular weight: 281.03 Number of HBA: 5 Number of HBD: 4 MolLogP: 0.92 MolLogS: −2.12 (in Log (moles/L)) 2121.05 (in mg/L) MolPSA: 65.75 A^2^ MolVol: 259.25 A^3^ Number of stereo centers: 1	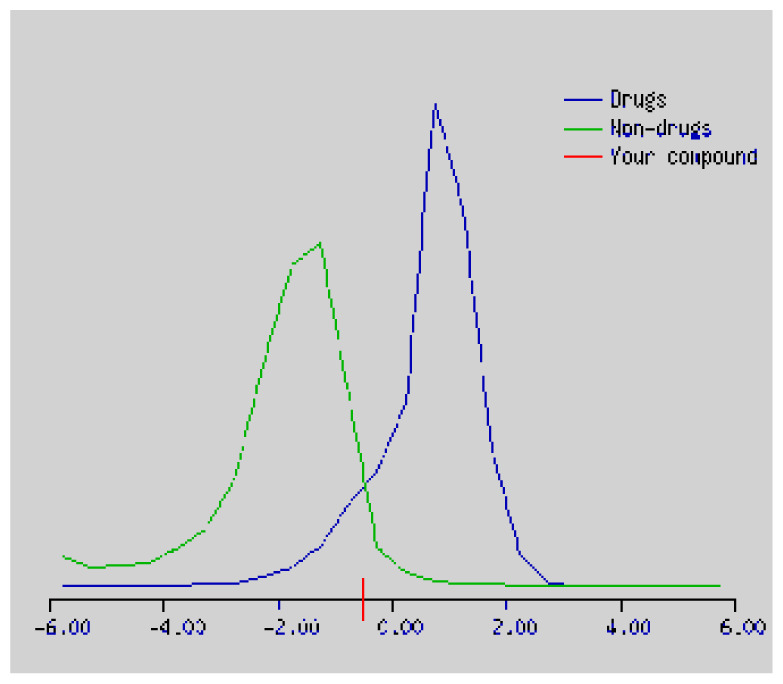 Drug-likeness model score: −0.50
**8**	C1=CC(=CC(=C1Cl)N(C(N(C(C(=O)O[H])CS[H])[H])=S)[H])OC	Molecular formula: C_11_H_14_ClN_2_O_3_S_2_ Molecular weight: 320.01 Number of HBA: 5 Number of HBD: 4 MolLogP: 1.88 MolLogS: −2.59 (in Log (moles/L)) 827.94 (in mg/L) MolPSA: 55.54 A^2^ MolVol: 273.58 A^3^ Number of stereo centers: 1	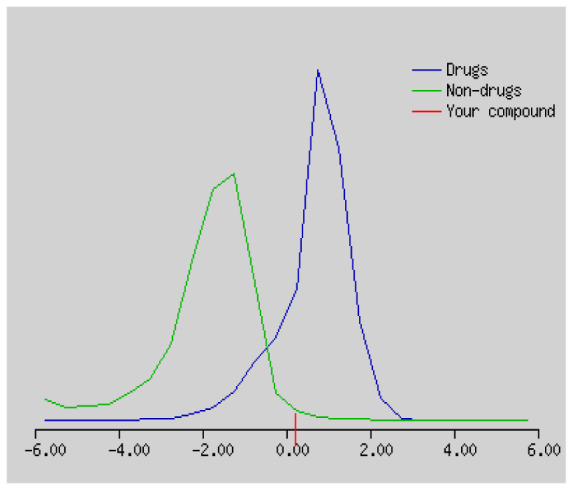 Drug-likeness model score: 0.23
**9**	C1=CC(=CC(=C1Cl)N(C(N(C(C(=O)O[H])CS[H])[H])=S)[H])OC(F)(F)F	Molecular formula: C_11_H_10_ClF_3_N_2_O_2_S_2_ Molecular weight: 357.98 Number of HBA: 4 Number of HBD: 4 MolLogP: 2.86 MolLogS: −3.23 (in Log (moles/L)) 209.66 (in mg/L) MolPSA: 48.00 A^2^ MolVol: 278.66 A^3^ Number of stereo centers: 1	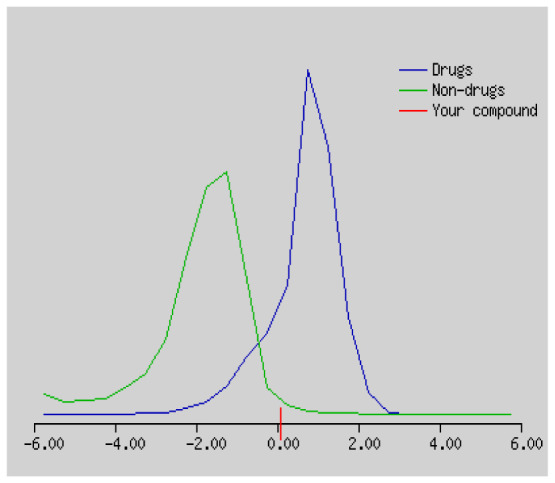 Drug-likeness model score: 0.07
**10**	C1=C(C=C(C(=C1)N(C(N(C(C(=O)O[H])CS[H])[H])=S)[H])OC)OC	Molecular formula: C_12_H_16_N_2_O_4_S_2_ Molecular weight: 316.06 Number of HBA: 6 Number of HBD: 4 MolLogP: 1.32 MolLogS: −2.12 (in Log (moles/L)) 2398.85 (in mg/L) MolPSA: 63.17 A^2^ MolVol: 289.44 A^3^ Number of stereo centers: 1	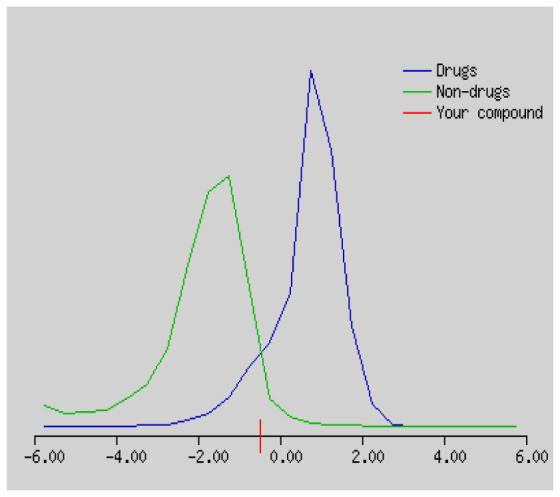 Drug-likeness model score: −0.49
**11**	C1(=CC=C(C(=C1)N(C(N(C(C(=O)O[H])CS[H])[H])=S)[H])OC)OC	Molecular formula: C_12_H_16_N_2_O_4_S_2_ Molecular weight: 316.06 Number of HBA: 6 Number of HBD: 4 MolLogP: 1.31 MolLogS: −2.12 (in Log (moles/L)) 2408.92 (in mg/L) MolPSA: 63.17 A^2^ MolVol: 289.44 A^3^ Number of stereo centers: 1	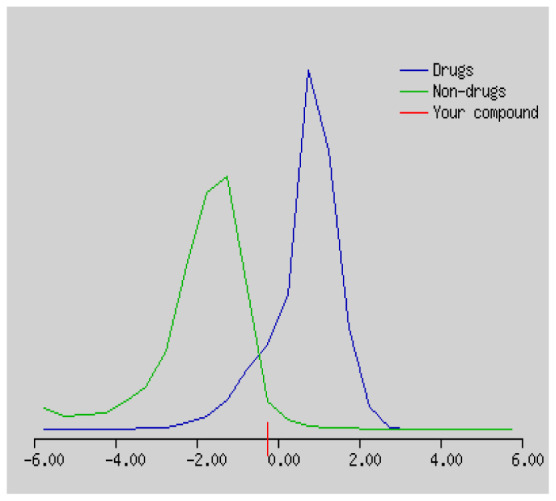 Drug-likeness model score: −0.26
**12**	C1=C(C(=CC(=C1)N(C(N(C(C(=O)O[H])CS[H])[H])=S)[H])OC)OC	Molecular formula: C_12_H_16_N_2_O_4_S_2_ Molecular weight: 316.06 Number of HBA: 6 Number of HBD: 4 MolLogP: 0.84 MolLogS: −2.00 (in Log (moles/L)) 3140.17 (in mg/L) MolPSA: 63.95 A^2^ MolVol: 288.55 A^3^ Number of stereo centers: 1	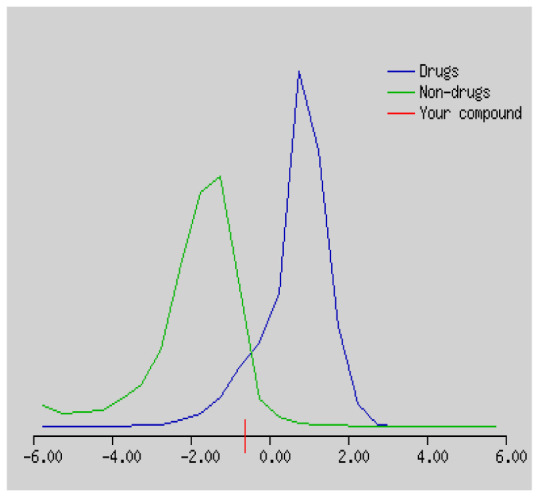 Drug-likeness model score: −0.62
**13**	C1(=CC(=CC(=C1)N(C(N(C(C(=O)O[H])CS[H])[H])=S)[H])OC)OC	Molecular formula: C_12_H_16_N_2_O_4_S_2_ Molecular weight: 316.06 Number of HBA: 6 Number of HBD: 4 MolLogP: 1.52 MolLogS: −2.06 (in Log (moles/L)) 2776.34 (in mg/L) MolPSA: 63.78 A^2^ MolVol: 289.19 A^3^ Number of stereo centers: 1	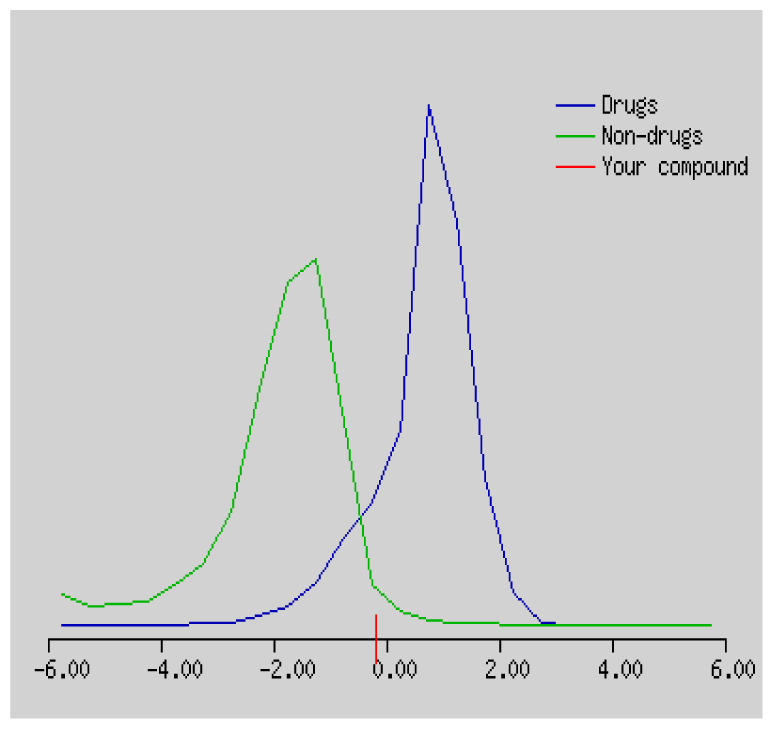 Drug-likeness model score: −0.20
**14**	C1(=C(C(=CC(=C1)N(C(N(C(C(=O)O[H])CS[H])[H])=S)[H])OC)OC)OC	Molecular formula: C_13_H_18_N_2_O_5_S_2_ Molecular weight: 346.07 Number of HBA: 7 Number of HBD: 4 MolLogP: 0.82 MolLogS: −2.08 (in Log (moles/L)) 2860.61 (in mg/L) MolPSA: 71.67 A^2^ MolVol: 320.39 A^3^ Number of stereo centers: 1	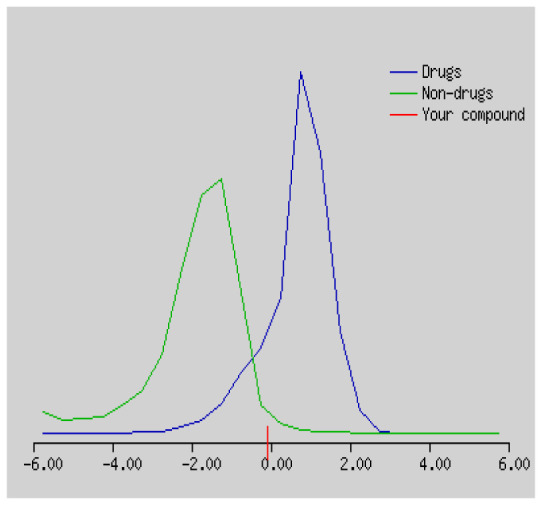 Drug-likeness model score: −0.07
**15**	C1=CC(=CC(=C1Cl)N(C(N(C(C(=O)O[H])CS[H])[H])=S)[H])Cl	Molecular formula: C_10_H_10_Cl_2_N_2_O_2_S_2_ Molecular weight: 323.96 Number of HBA: 4 Number of HBD: 4 MolLogP: 2.86 MolLogS: −3.14 (in Log (moles/L)) 235.51 (in mg/L) MolPSA: 48.69 A^2^ MolVol: 259.89 A^3^ Number of stereo centers: 1	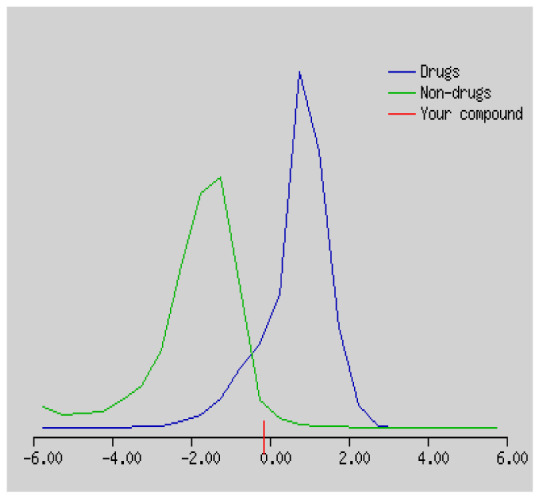 Drug-likeness model score: −0.14
**16**	C1=C(C=CC(=C1)N(C(N(C(C(=O)O[H])CS[H])[H])=S)[H])N(C)C	Molecular formula: C_12_H_17_N_3_O_2_S_2_ Molecular weight: 299.08 Number of HBA: 4 Number of HBD: 4 MolLogP: 1.38 MolLogS: −2.27 (in Log (moles/L)) 1594.71 (in mg/L) MolPSA: 51.50 A^2^ MolVol: 274.83 A^3^ Number of stereo centers: 1	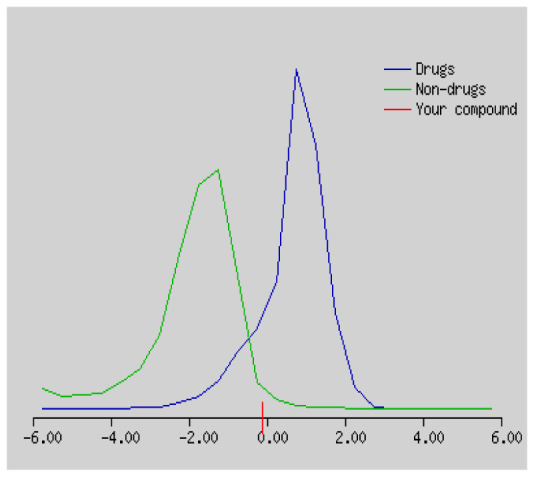 Drug-likeness model score: −0.13
**17**	C1=C(C=CC(=C1)N(C(N(C(C(=O)O[H])CS[H])[H])=S)[H])N(CC)CC	Molecular formula: C_14_H_21_N_3_O_2_S_2_ Molecular weight: 327.11 Number of HBA: 4 Number of HBD: 4 MolLogP: 2.12 MolLogS: −2.85 (in Log (moles/L)) 461245 (in mg/L) MolPSA: 51.43 A^2^ MolVol: 313.14 A^3^ Number of stereo centers: 1	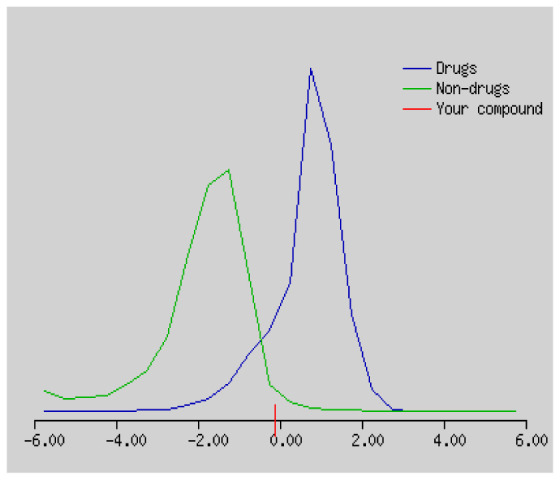 Drug-likeness model score: −0.11

**Table S2:** Predicted data of physicochemical characteristics, lipophilicity, solubility, pharmacokinetics, drug likeness, and medicinal chemistry of thioureas evaluated by SwissADME.

Thioureas	Physicochemical Properties	Lipophilicity	Water Solubility	Pharmacokinetics	Drug likeness	Medicinal Chemistry
**1**	Formula: C _10_H _12_N _2_O _2_S _2_ Mw: 256.03 g/mol Num. heavy atoms: 16 Num. arom. heavy atoms: 6 Fraction Csp3: 0.20 Num. rotatable bonds: 6 Num. HBA: 2 Num. HBD: 3 Molar Refractivity: 70.46 TPSA:132.25 Å ^2^	Log *P*o/w (iLOGP): 1.55 Log *P*o/w (XLOGP3): 1.44 Log *P*o/w (WLOGP): 1.17 Log *P*o/w (MLOGP): ^−1^.07 Log *P*o/w (SILICOS-IT): 1.72 Consensus Log *P*o/w: 0.96	Log *S* (ESOL): −2.22 Solubility: 1.55e-00 mg/mL; 6.05e-03 mol/L Class: Soluble Log *S* (Ali): −3.82 Solubility: 3.86e-02 mg/mL; 1.50e-04 mol/L Class: Soluble Log *S* (SILICOS-IT): −2.60 Solubility: 6.51e-01 mg/mL; 2.54e-03 mol/L Class: Soluble	GI absorption: High BBB permeant: No P-gp substrate: No CYP1A2 inhibitor: No CYP2C19 inhibitor: No CYP2C9 inhibitor: No CYP2D6 inhibitor: No CYP3A4 inhibitor: No Log *K*p (skin permeation): −6.84 cm/s	Lipinski: Yes Ghose: Yes Veber: Yes Egan: No Muegge: Yes Bioavailability Score: 0.56	PAINS: 0 alert Brenk: 2 alerts: thiocarbonyl_group, thiol_2 Leadlikeness: Yes Synthetic accessibility: 2.71
**2**	Formula: C _10_H _11_BrN _2_O _2_S _2_ Mw: 335.24 g/mol Num. heavy atoms: 17 Num. arom. heavy atoms: 6 Fraction Csp3: 0.20 Num. rotatable bonds: 6 Num. HBA: 2 Num. HBD: 3 Molar Refractivity: 78.16 TPSA:132.25 Å ^2^	Log *P*o/w (iLOGP): 2.17 Log *P*o/w (XLOGP3): 2.14 Log *P*o/w (WLOGP): 1.93 Log *P*o/w (MLOGP): −0.37 Log *P*o/w (SILICOS-IT): 2.43 Consensus Log *P*o/w: 1.66	Log *S* (ESOL): −3.13 Solubility: 2.47e-00 mg/mL; 7.38e-04 mol/L Class: Soluble Log *S* (Ali): −4.55 Solubility: 9.47e-03 mg/mL; 2.82e-05 mol/L Class: Moderately soluble Log *S* (SILICOS-IT): −3.41 Solubility: 1.30e-01 mg/mL; 3.86e-04 mol/L Class: Soluble	GI absorption: High BBB permeant: No P-gp substrate: No CYP1A2 inhibitor: Yes CYP2C19 inhibitor: No CYP2C9 inhibitor: No CYP2D6 inhibitor: No CYP3A4 inhibitor: No Log *K*p (skin permeation): −6.83 cm/s	Lipinski: Yes Ghose: Yes Veber: Yes Egan: No Muegge: Yes Bioavailability Score: 0.56	PAINS: 0 alert Brenk: 2 alerts: thiocarbonyl_group, thiol_2 Leadlikeness: Yes Synthetic accessibility: 2.82
**3**	Formula: C _10_H _11_ClN _2_O _2_S _2_ Mw: 290.79 g/mol Num. heavy atoms: 17 Num. arom. heavy atoms: 6 Fraction Csp3: 0.20 Num. rotatable bonds: 6 Num. HBA: 2 Num. HBD: 3 Molar Refractivity: 75.47 TPSA:132.25 Å ^2^	Log *P*o/w (iLOGP): 1.85 Log *P*o/w (XLOGP3): 2.07 Log *P*o/w (WLOGP): 1.82 Log *P*o/w (MLOGP): −0.51 Log *P*o/w (SILICOS-IT): 2.40 Consensus Log *P*o/w: 1.52	Log *S* (ESOL): −2.281 Solubility: 4.48e-01 mg/mL; 6.05e-03 mol/L Class: Soluble Log *S* (Ali): −4.48 Solubility: 9.71e-03 mg/mL; 3.34e-05 mol/L Class: Moderately soluble Log *S* (SILICOS-IT): −3.20 Solubility: 1.83e-01 mg/mL; 6.31e-04 mol/L Class: Soluble	GI absorption: High BBB permeant: No P-gp substrate: No CYP1A2 inhibitor: Yes CYP2C19 inhibitor: No CYP2C9 inhibitor: No CYP2D6 inhibitor: No CYP3A4 inhibitor: No Log *K*p (skin permeation): −6.60 cm/s	Lipinski: Yes Ghose: Yes Veber: Yes Egan: No Muegge: Yes Bioavailability Score: 0.56	PAINS: 0 alert Brenk: 2 alerts: thiocarbonyl_group, thiol_2 Leadlikeness: Yes Synthetic accessibility: 2.72
**4**	Formula: C _10_H _11_FN _2_O _2_S _2_ Mw: 274.33 g/mol Num. heavy atoms: 17 Num. arom. heavy atoms: 6 Fraction Csp3: 0.20 Num. rotatable bonds: 6 Num. HBA: 3 Num. HBD: 3 Molar Refractivity: 70.42 TPSA:132.25 Å ^2^	Log *P*o/w (iLOGP): 1.72 Log *P*o/w (XLOGP3): 1.54 Log *P*o/w (WLOGP): 1.72 Log *P*o/w (MLOGP): −0.66 Log *P*o/w (SILICOS-IT): 2.17 Consensus Log *P*o/w: 1.30	Log *S* (ESOL): −2.38 Solubility: 1.15e+00 mg/mL; 4205e-03 mol/L Class: Soluble Log *S* (Ali): −3.93 Solubility: 3.25e-02 mg/mL; 1.18e-04 mol/L Class: Soluble Log *S* (SILICOS-IT): −2.87 Solubility: 3.71e-01 mg/mL; 1.35e-03 mol/L Class: Soluble	GI absorption: High BBB permeant: No P-gp substrate: No CYP1A2 inhibitor: No CYP2C19 inhibitor: No CYP2C9 inhibitor: No CYP2D6 inhibitor: No CYP3A4 inhibitor: No Log *K*p (skin permeation): −6.88 cm/s	Lipinski: Yes Ghose: Yes Veber: Yes Egan: No Muegge: Yes Bioavailability Score: 0.56	PAINS: 0 alert Brenk: 2 alerts: thiocarbonyl_group, thiol_2 Leadlikeness: Yes Synthetic accessibility: 2.71
**5**	Formula: C _11_H _11_F _3_N _2_O _2_S _2_ Mw: 324.34 g/mol Num. heavy atoms: 20 Num. arom. heavy atoms: 6 Fraction Csp3: 0.27 Num. rotatable bonds: 7 Num. HBA: 5 Num. HBD: 3 Molar Refractivity: 75.46 TPSA:132.25 Å ^2^	Log *P*o/w (iLOGP): 1.95 Log *P*o/w (XLOGP3): 2.33 Log *P*o/w (WLOGP): 3.34 Log *P*o/w (MLOGP): −0.09 Log *P*o/w (SILICOS-IT): 2.88 Consensus Log Po/w: 2.08	Log *S* (ESOL): −3.08 Solubility: 2.71e-01 mg/mL; 8345e-04 mol/L Class: Soluble Log *S* (Ali): −4.75 Solubility: 3.86e-02 mg/mL; 1.50e-04 mol/L Class: Moderately soluble Log *S* (SILICOS-IT): −3.45 Solubility: 1.14e-01 mg/mL; 3.52e-04 mol/L Class: Soluble	GI absorption: High BBB permeant: No P-gp substrate: No CYP1A2 inhibitor: No CYP2C19 inhibitor: No CYP2C9 inhibitor: No CYP2D6 inhibitor: No CYP3A4 inhibitor: No Log *K*p (skin permeation): −6.62 cm/s	Lipinski: Yes Ghose: Yes Veber: Yes Egan: No Muegge: Yes Bioavailability Score: 0.56	PAINS: 0 alert Brenk: 2 alerts: thiocarbonyl_group, thiol_2 Leadlikeness: Yes Synthetic accessibility: 2.74
**6**	Formula: C _11_H _14_N _2_O _3_S _2_ Mw: 286.37 g/mol Num. heavy atoms: 18 Num. arom. heavy atoms: 6 Fraction Csp3: 0.27 Num. rotatable bonds: 7 Num. HBA: 3 Num. HBD: 3 Molar Refractivity: 76.95 TPSA:141.48 Å ^2^	Log *P*o/w (iLOGP): 1.85 Log *P*o/w (XLOGP3): 1.42 Log *P*o/w (WLOGP): 1.17 Log *P*o/w (MLOGP): −1.34 Log *P*o/w (SILICOS-IT): 1.80 Consensus Log *P*o/w: 0.98	Log *S* (ESOL): −2.29 Solubility: 1.45e+00 mg/mL; 5.07e-03 mol/L Class: Soluble Log *S* (Ali): −4.00 Solubility: 2.89e-02 mg/mL; 1.01e-04 mol/L Class: Soluble Log *S* (SILICOS-IT): −2.71 Solubility: 5.55e-01 mg/mL; 1.94e-03 mol/L Class: Soluble	GI absorption: High BBB permeant: No P-gp substrate: No CYP1A2 inhibitor: No CYP2C19 inhibitor: No CYP2C9 inhibitor: No CYP2D6 inhibitor: No CYP3A4 inhibitor: No Log *K*p (skin permeation): −7.04 cm/s	Lipinski: Yes Ghose: Yes Veber: No Egan: No Muegge: Yes Bioavailability Score: 0.56	PAINS: 0 alert Brenk: 2 alerts: thiocarbonyl_group, thiol_2 Leadlikeness: Yes Synthetic accessibility: 2.75
**7**	Formula: C _11_H _11_N _3_O _2_S _2_ Mw: 281.35 g/mol Num. heavy atoms: 18 Num. arom. heavy atoms: 6 Fraction Csp3: 0.18 Num. rotatable bonds: 6 Num. HBA: 3 Num. HBD: 3 Molar Refractivity: 75.18 TPSA:156.04 Å ^2^	Log *P*o/w (iLOGP): 1.55 Log *P*o/w (XLOGP3): 1.16 Log *P*o/w (WLOGP): 1.04 Log *P*o/w (MLOGP): −1.69 Log *P*o/w (SILICOS-IT): 1.78 Consensus Log *P*o/w: 0.77	Log *S* (ESOL): −2.17 Solubility: 1.92e+00 mg/mL; 6.83e-03 mol/L Class: Soluble Log *S* (Ali): −4.03 Solubility: 2.62e-02 mg/mL; 9.29e-05 mol/L Class: Moderately soluble Log *S* (SILICOS-IT): −2.67 Solubility: 5.96e-01 mg/mL; 2.12e-03 mol/L Class: Soluble	GI absorption: Low BBB permeant: No P-gp substrate: No CYP1A2 inhibitor: Yes CYP2C19 inhibitor: No CYP2C9 inhibitor: No CYP2D6 inhibitor: No CYP3A4 inhibitor: No Log *K*p (skin permeation): −7.19 cm/s	Lipinski: Yes Ghose: Yes Veber: No Egan: No Muegge: No Bioavailability Score: 0.11	PAINS: 0 alert Brenk: 2 alerts: thiocarbonyl_group, thiol_2 Leadlikeness: Yes Synthetic accessibility: 2.78
**8**	Formula: C _11_H _13_ClN _2_O _3_S _2_ Mw: 320.82 g/mol Num. heavy atoms: 19 Num. arom. heavy atoms: 6 Fraction Csp3: 0.27 Num. rotatable bonds: 7 Num. HBA: 3 Num. HBD: 3 Molar Refractivity: 81.96 TPSA:141.48 Å ^2^	Log *P*o/w (iLOGP): 2.05 Log *P*o/w (XLOGP3): 2.04 Log *P*o/w (WLOGP): 1.83 Log *P*o/w (MLOGP): −0.79 Log *P*o/w (SILICOS-IT): 2.48 Consensus Log *P*o/w: 1.52	Log *S* (ESOL): −2.89 Solubility: 4.17e-01 mg/mL; 1.30e-03 mol/L Class: Soluble Log *S* (Ali): −4.64 Solubility: 7.36e-03 mg/mL; 2.30e-05 mol/L Class: Moderately soluble Log *S* (SILICOS-IT): −3.31 Solubility: 1.56e-01 mg/mL; 4.86e-04 mol/L Class: Soluble	GI absorption: Low BBB permeant: No P-gp substrate: No CYP1A2 inhibitor: No CYP2C19 inhibitor: No CYP2C9 inhibitor: No CYP2D6 inhibitor: No CYP3A4 inhibitor: No Log *K*p (skin permeation): −6.81 cm/s	Lipinski: Yes Ghose: Yes Veber: No Egan: No Muegge: Yes Bioavailability Score: 0.56	PAINS: 0 alert Brenk: 2 alerts: thiocarbonyl_group, thiol_2 Leadlikeness: Yes Synthetic accessibility: 3.04
**9**	Formula: C _11_H _10_ClF _3_N _2_O _3_S _2_ Mw: 374.79 g/mol Num. heavy atoms: 22 Num. arom. heavy atoms: 6 Fraction Csp3: 0.27 Num. rotatable bonds: 8 Num. HBA: 6 Num. HBD: 3 Molar Refractivity: 82.16 TPSA:141.48 Å ^2^	Log *P*o/w (iLOGP): 2.30 Log *P*o/w (XLOGP3): 3.25 Log *P*o/w (WLOGP): 3.98 Log *P*o/w (MLOGP): −0.39 Log *P*o/w (SILICOS-IT): 3.15 Consensus Log *P*o/w: 2.46	Log *S* (ESOL): −3.88 Solubility: 4.88e-02 mg/mL; 1.30e-04 mol/L Class: Soluble Log *S* (Ali): −5.89 Solubility: 4.78e-04 mg/mL; 1.27e-06 mol/L Class: Moderately soluble Log *S* (SILICOS-IT): −3.78 Solubility: 6.28e-02 mg/mL; 1.67e-04 mol/L Class: Soluble	GI absorption: Low BBB permeant: No P-gp substrate: No CYP1A2 inhibitor: No CYP2C19 inhibitor: Yes CYP2C9 inhibitor: Yes CYP2D6 inhibitor: No CYP3A4 inhibitor: No Log *K*p (skin permeation): −6.28 cm/s	Lipinski: Yes Ghose: Yes Veber: No Egan: No Muegge: Yes Bioavailability Score: 0.56	PAINS: 0 alert Brenk: 2 alerts: thiocarbonyl_group, thiol_2 Leadlikeness: No Synthetic accessibility: 3.13
**10**	Formula: C _12_H _16_N _2_O _4_S _2_ Mw: 316.40 g/mol Num. heavy atoms: 20 Num. arom. heavy atoms: 6 Fraction Csp3: 0.33 Num. rotatable bonds: 8 Num. HBA: 4 Num. HBD: 3 Molar Refractivity: 83.45 TPSA:150.71 Å ^2^	Log *P*o/w (iLOGP): 2.11 Log *P*o/w (XLOGP3): 1.39 Log *P*o/w (WLOGP): 1.18 Log *P*o/w (MLOGP): −1.61 Log *P*o/w (SILICOS-IT): 1.89 Consensus Log Po/w: 0.99	Log *S* (ESOL): −2.37 Solubility: 1.35e+00 mg/mL; 4.25e-03 mol/L Class: Soluble Log *S* (Ali): −4.16 Solubility: 2.20e-02 mg/mL; 6.94e-05 mol/L Class: Moderately soluble Log *S* (SILICOS-IT): −2.83 Solubility: 4.71e-01 mg/mL; 1.49e-03 mol/L Class: Soluble	GI absorption: Low BBB permeant: No P-gp substrate: No CYP1A2 inhibitor: No CYP2C19 inhibitor: No CYP2C9 inhibitor: No CYP2D6 inhibitor: No CYP3A4 inhibitor: No Log *K*p (skin permeation): −7.24 cm/s	Lipinski: Yes Ghose: Yes Veber: No Egan: No Muegge: No Bioavailability Score: 0.11	PAINS: 0 alert Brenk: 2 alerts: thiocarbonyl_group, thiol_2 Leadlikeness: No Synthetic accessibility: 3.15
**11**	Formula: C _12_H _16_N _2_O _4_S _2_ Mw: 316.40 g/mol Num. heavy atoms: 20 Num. arom. heavy atoms: 6 Fraction Csp3: 0.33 Num. rotatable bonds: 8 Num. HBA: 4 Num. HBD: 3 Molar Refractivity: 83.45 TPSA:150.71 Å ^2^	Log *P*o/w (iLOGP): 1.96 Log *P*o/w (XLOGP3): 1.39 Log *P*o/w (WLOGP): 1.18 Log *P*o/w (MLOGP): −1.61 Log *P*o/w (SILICOS-IT): 1.89 Consensus Log *P*o/w: 0.96	Log *S* (ESOL): −2.37 Solubility: 1.35e+00 mg/mL; 4.25e-03 mol/L Class: Soluble Log *S* (Ali): −4.16 Solubility: 2.20e-02 mg/mL; 6.94e-05 mol/L Class: Moderately soluble Log *S* (SILICOS-IT): −2.83 Solubility: 4.71e-01 mg/mL; 1.49e-03 mol/L Class: Soluble	GI absorption: Low BBB permeant: No P-gp substrate: No CYP1A2 inhibitor: No CYP2C19 inhibitor: No CYP2C9 inhibitor: No CYP2D6 inhibitor: No CYP3A4 inhibitor: No Log *K*p (skin permeation): −7.24 cm/s	Lipinski: Yes Ghose: Yes Veber: No Egan: No Muegge: No Bioavailability Score: 0.11	PAINS: 0 alert Brenk: 2 alerts: thiocarbonyl_group, thiol_2 Leadlikeness: No Synthetic accessibility: 3.21
**12**	Formula: C _12_H _16_N _2_O _4_S _2_ Mw: 316.40 g/mol Num. heavy atoms: 20 Num. arom. heavy atoms: 6 Fraction Csp3: 0.33 Num. rotatable bonds: 8 Num. HBA: 4 Num. HBD: 3 Molar Refractivity: 83.45 TPSA:150.71 Å ^2^	Log *P*o/w (iLOGP): 2.01 Log *P*o/w (XLOGP3): 1.39 Log *P*o/w (WLOGP): 1.18 Log *P*o/w (MLOGP): −1.61 Log *P*o/w (SILICOS-IT): 1.89 Consensus Log *P*o/w: 0.97	Log *S* (ESOL): −2.37 Solubility: 1.35e+00 mg/mL; 4.25e-03 mol/L Class: Soluble Log *S* (Ali): −4.16 Solubility: 2.20e-02 mg/mL; 6.94e-05 mol/L Class: Moderately soluble Log *S* (SILICOS-IT): −2.83 Solubility: 4.71e-01 mg/mL; 1.49e-03 mol/L Class: Soluble	GI absorption: Low BBB permeant: No P-gp substrate: No CYP1A2 inhibitor: No CYP2C19 inhibitor: No CYP2C9 inhibitor: No CYP2D6 inhibitor: No CYP3A4 inhibitor: No Log *K*p (skin permeation): −7.24 cm/s	Lipinski: Yes Ghose: Yes Veber: No Egan: No Muegge: No Bioavailability Score: 0.11	PAINS: 0 alert Brenk: 2 alerts: thiocarbonyl_group, thiol_2 Leadlikeness: No Synthetic accessibility: 3.07
**13**	Formula: C _12_H _16_N _2_O _4_S _2_ Mw: 316.40 g/mol Num. heavy atoms: 20 Num. arom. heavy atoms: 6 Fraction Csp3: 0.33 Num. rotatable bonds: 8 Num. HBA: 4 Num. HBD: 3 Molar Refractivity: 83.45 TPSA:150.71 Å ^2^	Log *P*o/w (iLOGP): 2.23 Log *P*o/w (XLOGP3): 1.39 Log *P*o/w (WLOGP): 1.18 Log *P*o/w (MLOGP): −1.61 Log *P*o/w (SILICOS-IT): 1.89 Consensus Log *P*o/w: 1.02	Log *S* (ESOL): −2.37 Solubility: 1.35e-00 mg/mL; 4.25e-03 mol/L Class: Soluble Log *S* (Ali): −4.16 Solubility: 2.20e-02 mg/mL; 6.94e-05 mol/L Class: Moderately soluble Log *S* (SILICOS-IT): −2.83 Solubility: 4.71e-01 mg/mL; 1.49e-03 mol/L Class: Soluble	GI absorption: Low BBB permeant: No P-gp substrate: No CYP1A2 inhibitor: No CYP2C19 inhibitor: No CYP2C9 inhibitor: No CYP2D6 inhibitor: No CYP3A4 inhibitor: No Log *K*p (skin permeation): −7.24 cm/s	Lipinski: Yes Ghose: Yes Veber: No Egan: No Muegge: No Bioavailability Score: 0.11	PAINS: 0 alert Brenk: 2 alerts: thiocarbonyl_group, thiol_2 Leadlikeness: No Synthetic accessibility: 3.09
**14**	Formula: C _13_H _18_N _2_O _5_S _2_ Mw: 346.42 g/mol Num. heavy atoms: 22 Num. arom. heavy atoms: 6 Fraction Csp3: 0.38 Num. rotatable bonds: 9 Num. HBA: 5 Num. HBD: 3 Molar Refractivity: 89.94 TPSA:159.94 Å ^2^	Log *P*o/w (iLOGP): 2.21 Log *P*o/w (XLOGP3): 1.36 Log *P*o/w (WLOGP): 1.19 Log *P*o/w (MLOGP): −1.87 Log *P*o/w (SILICOS-IT): 1.99 Consensus Log *P*o/w: 0.98	Log *S* (ESOL): −2.45 Solubility: 1.22e+00 mg/mL; 3.53e-03 mol/L Class: Soluble Log *S* (Ali): −4.32 Solubility: 1.65e-02 mg/mL; 4.77e-05 mol/L Class: Moderately soluble Log *S* (SILICOS-IT): −2.94 Solubility: 4.01e-01 mg/mL; 1.16e-03 mol/L Class: Soluble	GI absorption: Low BBB permeant: No P-gp substrate: No CYP1A2 inhibitor: No CYP2C19 inhibitor: No CYP2C9 inhibitor: No CYP2D6 inhibitor: No CYP3A4 inhibitor: No Log *K*p (skin permeation): −7.45 cm/s	Lipinski: Yes Ghose: Yes Veber: No Egan: No Muegge: No Bioavailability Score: 0.11	PAINS: 0 alert Brenk: 2 alerts: thiocarbonyl_group, thiol_2 Leadlikeness: No Synthetic accessibility: 3.27
**15**	Formula: C _10_H _10_ClN _2_O _2_S _2_ Mw: 325.23 g/mol Num. heavy atoms: 18 Num. arom. heavy atoms: 6 Fraction Csp3: 0.20 Num. rotatable bonds: 6 Num. HBA: 2 Num. HBD: 3 Molar Refractivity: 80.48 TPSA:132.25 Å ^2^	Log *P*o/w (iLOGP): 2.14 Log *P*o/w (XLOGP3): 2.70 Log *P*o/w (WLOGP): 2.47 Log *P*o/w (MLOGP): 0.04 Log *P*o/w (SILICOS-IT): 3.07 Consensus Log *P*o/w: 2.08	Log *S* (ESOL): −3.41 Solubility: 1.27e-01 mg/mL; 3.91e-04 mol/L Class: Soluble Log *S* (Ali): −5.13 Solubility: 2.41e-0 mg/mL; 7.41e-06 mol/L Class: Moderately soluble Log *S* (SILICOS-IT): −3.80 Solubility: 5.17e-02 mg/mL; 1.59e-04 mol/L Class: Soluble	GI absorption: High BBB permeant: No P-gp substrate: No CYP1A2 inhibitor: Yes CYP2C19 inhibitor: No CYP2C9 inhibitor: Yes CYP2D6 inhibitor: No CYP3A4 inhibitor: No Log *K*p (skin permeation): −6.37 cm/s	Lipinski: Yes Ghose: Yes Veber: Yes Egan: No Muegge: Yes Bioavailability Score: 0.56	PAINS: 0 alert Brenk: 2 alerts: thiocarbonyl_group, thiol_2 Leadlikeness: Yes Synthetic accessibility: 2.92
**16**	Formula: C _12_H _17_N _3_O _2_S _2_ Mw: 299.41 g/mol Num. heavy atoms: 19 Num. arom. heavy atoms: 6 Fraction Csp3: 0.33 Num. rotatable bonds: 7 Num. HBA: 2 Num. HBD: 3 Molar Refractivity: 84.67 TPSA:135.49 Å ^2^	Log *P*o/w (iLOGP): 1.93 Log *P*o/w (XLOGP3): 1.57 Log *P*o/w (WLOGP): 1.23 Log *P*o/w (MLOGP): −1.06 Log *P*o/w (SILICOS-IT): 1.42 Consensus Log *P*o/w: 1.02	Log *S* (ESOL): −2.46 Solubility: 1.05e+00 mg/mL; 3.49e-03 mol/L Class: Soluble Log *S* (Ali): −4.03 Solubility: 2.82e-02 mg/mL; 9.43e-05 mol/L Class: Moderately soluble Log *S* (SILICOS-IT): −2.69 Solubility: 6.10e-01 mg/mL; 2.04e-03 mol/L Class: Soluble	GI absorption: High BBB permeant: No P-gp substrate: No CYP1A2 inhibitor: No CYP2C19 inhibitor: No CYP2C9 inhibitor: No CYP2D6 inhibitor: No CYP3A4 inhibitor: No Log *K*p (skin permeation): −7.01 cm/s	Lipinski: Yes Ghose: Yes Veber: Yes Egan: No Muegge: Yes Bioavailability Score: 0.56	PAINS: 1 alert Brenk: 2 alerts: thiocarbonyl_group, thiol_2 Leadlikeness: Yes Synthetic accessibility: 2.97
**17**	Formula: C _14_H _21_N _3_O _2_S _2_ Mw: 327.47 g/mol Num. heavy atoms: 21 Num. arom. heavy atoms: 6 Fraction Csp3: 0.43 Num. rotatable bonds: 9 Num. HBA: 2 Num. HBD: 3 Molar Refractivity: 94.28 TPSA:135.49 Å ^2^	Log *P*o/w (iLOGP): 2.37 Log *P*o/w (XLOGP3): 2.30 Log *P*o/w (WLOGP): 2.01 Log *P*o/w (MLOGP): −0.53 Log *P*o/w (SILICOS-IT): 2.21 Consensus Log *P*o/w: 1.67	Log *S* (ESOL): −2.94 Solubility: 3.79e-00 mg/mL; 1.16e-03 mol/L Class: Soluble Log *S* (Ali): −4.78 Solubility: 5.39e-03 mg/mL; 1.65e-05 mol/L Class: Moderately soluble Log *S* (SILICOS-IT): −3.49 Solubility: 1.07e-01 mg/mL; 3.27e-04 mol/L Class: Soluble	GI absorption: High BBB permeant: No P-gp substrate: No CYP1A2 inhibitor: No CYP2C19 inhibitor: No CYP2C9 inhibitor: No CYP2D6 inhibitor: No CYP3A4 inhibitor: No Log *K*p (skin permeation): −6.66 cm/s	Lipinski: Yes Ghose: Yes Veber: Yes Egan: No Muegge: Yes Bioavailability Score: 0.56	PAINS: 1 alert Brenk: 2 alerts: thiocarbonyl_group, thiol_2 Leadlikeness: No Synthetic accessibility: 3.14

**Table S3:** Molinspiration bioactivity scores and Molsoft calculated percentage absorptions for thioureas **1–17**.

Thioureas	Molinspiration bioactivity scores						Molsoft
	GPCRL	ICM	KI	NRL	PI	EI	Absorption, %
**1**	−0.53	−0.35	−0.94	−0.97	−0.07	0.19	87.83
**2**	−0.59	−0.42	−0.89	−1.00	−0.18	0.11	87.83
**3**	−0.45	−0.32	−0.85	−0.87	−0.07	0.17	87.83
**4**	−0.43	−0.33	−0.78	−0.81	−0.04	0.19	87.83
**5**	−0.16	−0.15	−0.48	−0.37	0.18	0.25	87.83
**6**	−0.43	−0.39	−0.77	−0.75	−0.03	0.17	84.65
**7**	−0.34	−0.29	−0.59	−0.60	0.07	0.29	79.63
**8**	−0.41	−0.40	−0.66	−0.76	−0.12	0.08	84.65
**9**	−0.16	−0.17	−0.41	−0.39	0.10	0.15	87.83
**10**	−0.32	−0.43	−0.60	−0.60	−0.02	0.14	81.46
**11**	−0.33	−0.41	−0.59	−0.64	−0.01	0.13	81.46
**12**	−0.32	−0.38	−0.57	−0.65	0.02	0.16	81.46
**13**	−0.30	−0.36	−0.58	−0.58	0.05	0.18	81.46
**14**	−0.24	−0.34	−0.46	−0.59	0.08	0.16	78.27
**15**	−0.39	−0.30	−0.71	−0.84	−0.10	0.13	87.83
**16**	−0.28	−0.29	−0.59	−0.61	0.08	0.23	86.72
**17**	−0.15	−0.28	−0.50	−0.49	0.13	0.17	86.72

GPCRL: GPCR ligand; ICM: Ion channel modulator; KI: Kinase inhibitor; NRL: Nuclear receptor ligend; PI: Protease inhibitor; EI: Enzyme inhibitor.

## Figures and Tables

**Table 1:** ^1^H NMR spectra data of synthesized thiourea derivatives **(1 – 17)**.

Compound	^1^H NMR (400 MHz, DMSO - *d_6_*)
**1**	10.01 (s, 1H, - O**H**), 7.97 (s, 1H, Ar -N**H**CSNH - CH), 7.53 (d, *J* = 8.0 Hz, 2H, *ortho* protons of Ar - NHCSNH - CH), 7.34 - 7.32 (m, 3H, *meta* protons of Ar - NHCSNH - CH and Ar – NHCSN**H** - CH), 7.13 (t, *J_1_* = 6.8, *J_2_* = 6.0 Hz, 1H, *para* protons of Ar - NHCSNH - C**H**), 5.16 (m, 1H, Ar - NHCSNH - C**H**), 3.70 (dd, *J_1_* = 4.8, *J_2_* = 4 Hz, 1H, - CHCH_2_S**H**), 3.10 (dd, *J_1_* = 5.2, *J_2_* = 4.0 Hz, 1H, - CHC**H_2_**SH, **H_a_**), 2.99 (dd, *J_1_* = 5.2, *J_2_* = 4.8 Hz, 1H, -CHC**H_2_**SH, **H_b_**).
**2**	9.95 (s, 1H, - O**H**), 7.94 (s, 1H, Ar - N**H**CSNH - CH), 7.18 (d, *J* = 7.6 Hz, 2H, *ortho* protons of Ar - NHCSNH - CH), 7.55 - 7.43 (m, 3H, *meta* protons of Ar - NHCSNH - CH and Ar - NHCSN**H** - CH), 5.15 (m, 1H, Ar – NHCSNH - C**H**), 3.09 (dd, *J_1_* = 9.6, *J_2_* = 4.0 Hz, 1H, - CHCH_2_S**H**), 2.98 (dd, *J_1_* = 9.6, *J_2_* = 4.0 Hz, 1H, - CHC**H_2_**SH, **H_a_**), 2.38 (dd, *J_1_*= 4.0 Hz, *J_2_* = 4.0 Hz, 1H, - CHC**H_2_**SH, **H_b_**).
**3**	10.12 (s, 1H, - O**H**), 8.15 (s, 1H, Ar - N**H**CSNH - CH), 7.47 - 7.45 (m, 3H, *meta* protons of Ar - NHCSNH - CH and Ar - NHCSN**H** - CH), 7.37 (d, *J*= 8.0 Hz, 2H, *ortho* protons of Ar - NHCSNH - CH), 5.10 (m, 1H, Ar - NHCSNH - C**H**), 3.97 (dd, *J_1_* = 5.6, *J_2_*=4.2 Hz, 1H, - CHCH_2_S**H**), 2.91 (dd, *J_1_*=5.8, *J_2_* = 5.6 Hz, 1H, - CHC**H_2_**SH, **H_a_**), 2.82 (dd, *J_1_* = 5.6, *J_2_* = 4.2 Hz, 1H, - CHC**H_2_**SH, **H_b_**).
**4**	10.44 (s, 1H, - O**H**), 7.94 (s, 1H, Ar - N**H**CSNH - CH), 7.82 (s, 1H, Ar - NHCSN**H** - CH), 7.0 (m, 2H, *meta* protons of Ar - NHCSNH - CH), 6.78 (t, *J_1_* = 8.4, *J_2_* = 8.8 Hz, 2H, *ortho* protons of Ar - NHCSNH - CH), 5.08 - 4.94 (m, 1H, Ar - NHCSNH - C**H**), 3.64 (dd, *J_1_* = 8.8, *J_2_* = 4.8 Hz, 1H, -CHC**H_2_**SH, **H_a_**), 3.19 (dd, *J_1_* = 8.8, *J_2_* = 4.2 Hz, 1H, - CHC**H_2_**SH, **H_b_**), 3.05 (dd, *J_1_* = 4.8, *J_2_* = 4.2 Hz, 1H, - CHCH_2_S**H**).
**5**	9.85 (s, 1H, - O**H**), 8.76 (s, 1H, Ar - N**H**CSNH - CH), 7.83 (d, *J* = 8.4 Hz, 2H, *meta* protons of Ar - NHCSNH - CH), 7.79 (s, 1H, Ar - NHCSN**H** - CH), 7.46 (d, *J* = 7.2 Hz, 2H, *ortho* protons of Ar - NHCSNH - CH), 5.15 (m, 1H, Ar - NHCSNH - C**H**), 3.89 (dd, *J_1_* = 4.6, *J_2_* = 4.0 Hz, 1H, - CHCH_2_S**H**), 2.11 (dd, *J_1_* = 5.8, *J_2_* = 4.6 Hz, 1H, - CHC**H_2_**SH, **H_a_**), 2.71 (dd, *J_1_* = 5.2, *J_2_* = 4.0 Hz, 1H, - CHC**H_2_**SH, **H_b_**).
**6**	9.96 (s, 1H, - O**H**), 7.78 (s, 1H, Ar - N**H**CSNH - CH), 7.56 (s, 1H, Ar - NHCSN**H** - CH), 7.35 (d, *J* = 8.0 Hz, 2H, *meta* protons of Ar - NHCSNH - CH), 6.92 (d, *J* = 8.0 Hz, 2H, *ortho* protons of Ar - NHCSNH - CH), 5.12 (m, 1H, Ar - NHCSNH - C**H**), 3.65 (dd, *J_1_* = 4.8, *J_2_* = 4.2 Hz, 1H, - CHCH_2_S**H**), 3.44 (s, 3H, - OCH_3_), 2.97 (dd, *J_1_* = 5.4, *J_2_* = 4.8 Hz, 1H, - CHC**H_2_**SH, **H_a_**), 2.90 (dd, *J_1_* = 5.8, *J_2_* = 4.2 Hz, 1H, - CHC**H_2_**SH, **H_b_**).
**7**	10.42 (s, 1H, - O**H**), 8.47 (s, 1H, Ar - N**H**CSNH - CH), 7.87 (s, 1H, Ar - NHCSN**H** - CH), 7.22 (d, *J* = 6.8 Hz, 2H, *meta* protons of Ar - NHCSNH - CH), 6.54 (d, *J* = 6.8 Hz, 2H, *ortho* protons of Ar - NHCSNH - CH), 5.29 (m, 1H, Ar - NHCSNH - C**H**), 3.46 (dd, *J_1_* = 5.2, *J_2_* = 4.6 Hz, 1H, - CHCH_2_S**H**), 3.10 (dd, *J_1_* = 5.8, *J_2_* = 5.2 Hz, 1H, - CHC**H_2_**SH, **H_a_**), 2.99 (dd, *J_1_* = 5.6, *J_2_* = 4.6 Hz, 1H, - CHC**H_2_**SH, **H_b_**).
**8**	11.47 (s, 1H, - O**H**), 10.55 (s, 1H, Ar - **NH**CSNH - CH), 8.60 (s, 1H, Ar - NHCS**NH** - CH), 7.67 (s, 1H, **Ar - H**), 7.26 - 7.46 (m, 2H, Ar **- H**), 5.17 - 5.10 (m, 1H, - C**H**CH_2_SH), 3.98 (s, 3H, OC**H_3_**), 3.12 (dd, *J_1_* = 5.8, *J_2_* = 5.6 Hz, 1H, - CHC**H_2_**SH, **H_a_**), 3.08 (dd, *J_1_* = 5.8, *J_2_* = 4.2 Hz, 1H, - CHC**H_2_**SH, **H_b_**), 2.97 (dd, *J_1_* = 5.6, *J_2_* = 4.0 Hz, 1H, - CHCH_2_S**H**).
**9**	10.21 (s, 1H, - O**H**), 9.07 (s, 1H, Ar - **NH**CSNH - CH), 7.13 - 6.99 (m, 2H, Ar - NHCS**NH** - CH and Ar - **H**), 6.60 (d, *J* = 8.3 Hz, 2H, Ar - **H**), 4.75 - 4.62 (m, 1H, - C**H**CH_2_SH), 3.33 - 3.36 (m, 2H, - CHC**H_2_**SH, **H_a_** and **H_b_**), 1.08 (dd, *J_1_* = 5.2, *J_2_* = 4.2 Hz, 1H, - CHCH_2_S**H**).
**10**	11.57 (s, 1H, - O**H**), 9.68 (s, 1H, Ar - **NH**CSNH - CH), 9.61 (s, 1H, Ar - NHCS**NH** - CH), 6.00 - 5.91 (m, 3H, **Ar - H**), 5.16 - 5.06 (m, 1H, - C**H**CH_2_SH), 3.95 (s, 6H, OC**H_3_**), 2.81-2.95 (m, 2H, - CHC**H_2_**SH, **H_a_** and **H_b_**), 2.33 (dd, *J_1_* = 4.8, *J_2_* = 4.0 Hz, 1H, -CHCH_2_S**H**).
**11**	10.99 (s, 1H, - O**H**), 9.76 (s, 1H, Ar - **NH**CSNH - CH), 9.55 (s, 1H, Ar - NHCS**NH** - CH), 6.67 - 7.01 (m, 3H, **Ar - H**), 5.14 - 5.07 (m, 1H, - C**H**CH_2_SH), 3.83 (s, 6H, OC**H_3_**), 2.87 – 3.03 (m, 2H, - CHC**H_2_**SH, **H_a_** and **H_b_**), 2.38 (dd, *J_1_* = 4.8, *J_2_* = 4.2 Hz, 1H, - CHCH_2_S**H**).
**12**	11.66 (s, 1H, - O**H**), 9.88 (s, 1H, Ar - **NH**CSNH - CH), 9.69 (s, 1H, Ar - NHCS**NH** - CH), 6.80 – 7.14 (m, 3H, **Ar - H**), 5.13 - 5.08 (m, 1H, - C**H**CH_2_SH), 3.70 (s, 6H, OC**H_3_**), 2.98 – 3.10 (m, 2H, - CHC**H_2_**SH, **H_a_** and **H_b_**), 2.41 (dd, *J_1_* = 5.0, *J_2_* = 4.4 Hz, 1H, - CHCH_2_S**H**).
**13**	10.68 (s, 1H, - O**H**), 8.95 (s, 1H, Ar - **NH**CSNH - CH), 8.47 (s, 1H, Ar - NHCS**NH** - CH), 7.24 - 6.70 (m, 3H, **Ar - H**), 5.16 - 5.11 (m, 1H, - C**H**CH_2_SH), 3.78 (s, 6H, OC**H_3_**), 2.92 – 3.02 (m, 2H, - CHC**H_2_**SH, **H_a_** and **H_b_**), 2.33 (dd, *J_1_* = 5.6, *J_2_* = 4.8 Hz, 1H, - CHCH_2_S**H**).
**14**	10.34 (s, 1H, - O**H**), 9.42 (s, 1H, Ar - **NH**CSNH - CH), 8.45 (s, 1H, Ar – NHCS**NH** - CH), 6.97 - 6.24 (m, 2H, **Ar - H**), 5.17 - 5.12 (m, 1H, - C**H**CH_2_SH), 3.76 (s, 9H, OC**H_3_**), 3.38 - 3.17 (m, 2H, - CHC**H_2_**SH, **H_a_** and **H_b_**), 2.40 (dd, *J_1_* = 5.0, *J_2_* = 4.6 Hz, 1H, - CHCH_2_S**H**).
**15**	11.00 (s, 1H, - O**H**), 9.85 (s, 1H, Ar - **NH**CSNH - CH), 9.63 (s, 1H, Ar - NHCS**NH** - CH), 7.09 - 6.66 (m, 3H, **Ar - H**), 5.16 - 5.10 (m, 1H, - C**H**CH_2_SH), 3.79-3.64 (m, 2H, -CHC**H_2_**SH, **H_a_** and **H_b_**), 3.17 (dd, *J_1_*= 5.2, *J_2_*= 4.8 Hz, 1H, -CHCH_2_S**H**).
**16**	11.72 (s, 1H, - O**H**), 9.43 (s, 1H, Ar - **NH**CSNH - CH), 9.02 (s, 1H, Ar - NHCS**NH** - CH), 7.05 - 6.64 (m, 4H, **Ar - H**), 5.38 - 5.29 (m, 1H, - C**H**CH_2_SH), 3.14 (dd, *J_1_* = 5.2, *J_2_* = 4.0 Hz, 1H, - CHC**H_2_**SH, **H_a_**), 3.06 (dd, *J_1_* = 5.2, *J_2_* = 4.8 Hz, 1H, - CHC**H_2_**SH, **H_b_**), 2.93 (s, 6H, - N(C**H_3_**)**_2_**), 2.87 (dd, *J_1_* = 4.8, *J_2_* = 4.0 Hz, 1H, - CHCH_2_S**H**).
**17**	11.69 (s, 1H, - O**H**), 9.86 (s, 1H, Ar - **NH**CSNH - CH), 9.68 (s, 1H, Ar - NHCS**NH** - CH), 7.78 - 7.26 (m, 2H, **Ar - H**), 6.96 - 6.68 (m, 2H, **Ar - H**), 5.38 - 5.27 (m, 1H, - C**H**CH_2_SH), 3.76 - 3.62 (m, 4H, N(C**H_2_**CH_3_)**_2_**), 3.12 (dd, *J_1_* = 5.6, *J_2_* = 4.0 Hz, 1H, - CHC**H_2_**SH, **H_a_**), 2.99 (dd, *J_1_* = 5.6, *J_2_* = 4.6 Hz, 1H, - CHC**H_2_**SH, **H_b_**), 2.84 (dd, *J_1_* =4.6, *J_2_* = 4.0 Hz, 1H, - CHCH_2_S**H**), 1.32 (s, 6H, N(CH_2_C**H_3_**)**_2_**).

**Table 2:** Anticholinesterase, tyrosinase and urease inhibitory activities of the synthesized compounds **(1–17)**^a^.

Compound	Anticholinesterase Inhibitory Activity		Tyrosinase Inhibitory Activity	Urease Inhibitory Activity
	AChE assay IC_50_ (μM)	BChE assay IC_50_ (μM)	Tyrosinase assay IC_50_ (mM)	Urease assay IC_50_ (μM)
**1**	23.4 ± 1.2	66.0 ± 0.1	9.7 ± 1.0	64.6 ± 0.4
**2**	36.4 ± 0.2	75.1 ± 0.5	28.8 ± 0.1	66.1 ± 0.7
**3**	31.7 ± 0.8	72.8 ± 1.1	16.1 ± 0.9	58.2 ± 0.4
**4**	28.5 ± 0.5	70.4 ± 0.9	12.5 ± 0.3	61.5 ± 0.3
**5**	16.7 ± 0.2	64.3 ± 0.7	10.4 ± 0.2	46.2 ± 0.7
**6**	8.1 ± 0.9	58.2 ± 0.1	1.9 ± 0.5	31.3 ± 0.2
**7**	46.8 ± 1.3	84.4 ± 0.9	35.0 ± 1.1	68.3 ± 0.5
**8**	9.5 ± 1.2	61.2 ± 0.5	4.0 ± 0.6	34.8 ± 0.2
**9**	40.3 ± 1.4	80.0 ± 0.6	15.7 ± 0.5	50.2 ± 0.1
**10**	6.8 ± 1.1	45.8 ± 0.4	1.5 ± 0.3	20.9 ± 1.0
**11**	7.2 ± 0.5	49.7 ± 0.9	2.9 ± 0.2	22.1 ± 0.1
**12**	7.8 ± 0.6	54.0 ± 1.3	1.6 ± 0.6	26.5 ± 0.4
**13**	5.7 ± 1.0	37.2 ± 0.1	2.2 ± 0.4	16.5 ± 0.6
**14**	4.8 ± 0.9	29.5 ± 1.1	1.1 ± 0.1	13.4 ± 0.8
**15**	44.6 ± 0.3	82.9 ± 0.4	22.2 ± 0.9	55.4 ± 1.3
**16**	4.2 ± 0.6	24.6 ± 0.7	4.7 ± 0.3	44.3 ± 1.1
**17**	3.9 ± 0.6	18.1 ± 0.5	5.3 ± 0.8	38.0 ± 0.4
**Galantamine** b	4.6 ± 0.1	46.4 ± 0.8	NT	NT
**Kojic acid** b	NT	NT	0.66 ± 0.4	NT
**L-mimosine** b	NT	NT	0.70 ± 0.1	NT
**Thiourea** b	NT	NT	NT	24.20 ± 0.3

a Values expressed are means ± S.E.M. of three parallel measurements. *p*< 0.05, significantly different with student’s *t*-test.

b Reference compounds.

NT: Not tested.
