# Supplementary figures and images for: Synthesis, predictions of drug-likeness, and pharmacokinetic properties of some chiral thioureas as potent enzyme inhibition agents
Source: Turk J Chem. 2021 Sep 16;46(3):665–76. doi: 10.55730/1300-0527.3358 (PMC10503972; doi:10.55730/1300-0527.3358)

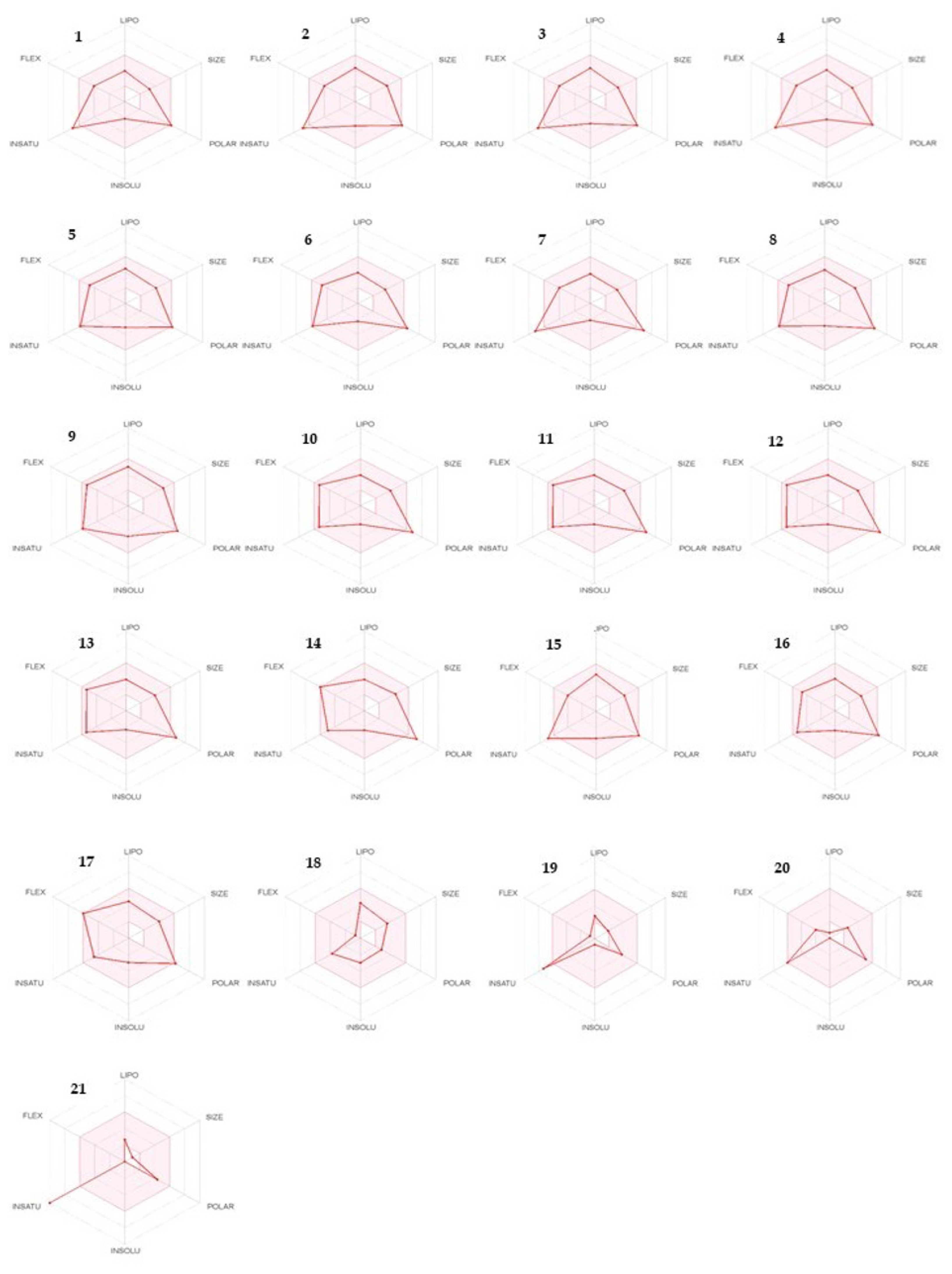

Supplement: Figure S1 — The bioavailability radar of synthesized chiral thioureas and enzyme inhibitor standards using Swiss ADME predictor. [file turkjchem-46-3-665s1.tif]
